# Antipsychotics possess anti-glioblastoma activity by disrupting lysosomal function and inhibiting oncogenic signaling by stabilizing PTEN

**DOI:** 10.1038/s41419-024-06779-3

**Published:** 2024-06-13

**Authors:** John Ryan Jacob, Kamalakannan Palanichamy, Arnab Chakravarti

**Affiliations:** Department of Radiation Oncology, The Ohio State University College of Medicine and Comprehensive Cancer Center, Columbus, OH 43210 USA

**Keywords:** CNS cancer, Chaperone-mediated autophagy

## Abstract

The repurposing of medications developed for central nervous system (CNS) disorders, possessing favorable safety profiles and blood-brain barrier permeability, represents a promising strategy for identifying new therapies to combat glioblastoma (GBM). In this study, we investigated the anti-GBM activity of specific antipsychotics and antidepressants in vitro and in vivo. Our results demonstrate that these compounds share a common mechanism of action in GBM, disrupting lysosomal function and subsequently inducing lysosomal membrane rupture and cell death. Notably, *PTEN* intact GBMs possess an increased sensitivity to these compounds. The inhibition of lysosomal function synergized with inhibitors targeting the EGFR-PI3K-Akt pathway, leading to an energetic and antioxidant collapse. These findings provide a foundation for the potential clinical application of CNS drugs in GBM treatment. Additionally, this work offers critical insights into the mechanisms and determinants of cytotoxicity for drugs currently undergoing clinical trials as repurposing agents for various cancers, including Fluoxetine, Sertraline, Thioridazine, Chlorpromazine, and Fluphenazine.

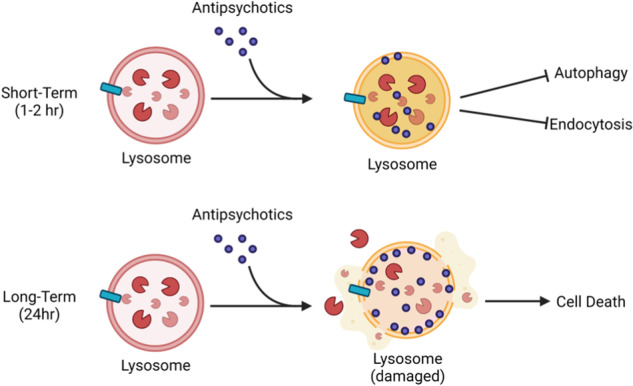

## Introduction

Glioblastoma (GBM) is one of the deadliest human cancers, with an average survival rate between 10 and 15 months [1]. This highlights the urgent need for new therapeutic approaches that can be rapidly implemented in clinical settings. One promising strategy is the repurposing of FDA-approved antipsychotics. These drugs are attractive candidates for repurposing due to their established safety profiles and ability to penetrate the blood–brain barrier (BBB) [2]. Antipsychotics are reported to possess an inverse relationship with cancer incidence and have been linked with various anti-cancer properties in vitro [3–11]. GBM patients are often prescribed antipsychotics to manage their comorbid psychiatric conditions [12], indicating their safe use in this population. Despite these promising indications, the efficacy, and underlying mechanisms of action of antipsychotics in GBM remain largely unexplored.

In this study, we performed cytotoxicity screens to identify first and second-generation antipsychotics that possess anti-GBM activity. We report the previously unknown mechanism of antipsychotic cytotoxicity in GBMs whereby accumulation in lysosomes disrupts catabolic function, suppressing autophagic and endocytic flux. Over time, this accumulation leads to lysosomal membrane permeability and cell death. *PTEN* intact GBMs possess an enhanced sensitivity to antipsychotics, corresponding with the simultaneous inhibition of Akt/mTORC1 activation and lysosomal function. Interestingly in *PTEN* mutant GBMs, combining EGFR and Akt inhibitors enhances antipsychotic sensitivity, leading to a collapse of cellular redox potential and energetics. We conclude that the antipsychotics identified in this study possess promising repurposing potential in GBM by disrupting lysosomal function and oncogenic kinase signaling.

## Materials and methods

### Study approval

This study was conducted in accordance with The Ohio State University Intuitional Review Boards for IRB (2009C0065 and 2014C0115), IACUC (2009A0127), and IBC (2009R0169). Informed consent was obtained from all subjects.

### Cell lines and tissue culture

U87-MG (HTB-14), U87-MG-Luc2 (HTB-14-LUC2), A172 (CRL-1620), T98G (CRL-1690) U118-MG (HTB-15), and LN18 (CRL-2610) were purchased from American Type Culture Collective. OSU2, OSU61, ACPK1, ACPK4 and ACPK8 were isolated from GBM tissue. GBM cell lines were cultured in DMEM, high glucose, pyruvate (Thermo: 11995073) supplemented with 10% FBS (Invitrogen) and 1% antibiotic–antimycotic (Thermo: 15240062). GSC lines OSU61, OSU11, OSU17, ACPK1, ACPK4 and ACPK8 were grown as neurospheres and cultured in DMEM-F12 (Invitrogen) supplemented with B-27 (Thermo Fisher: 17504044), 20 ng/mL EGF (Thermo Fisher: PHG0311), 20 ng/mL bFGF (Thermo Fisher: PHG0369), and 1% antibiotic–antimycotic (Thermo Fisher: 13256-029). The basic tumor characteristics of the cell lines first used in this study are as follows: ACPK1 (GBM-Recurrent, female, 26 yr, IDH1/2 status – NA), ACPK4 (GBM, male, 84 yr, IDH1/2 status - WT), and ACPK8 (GBM-Recurrent, male, 36 yr, IDH1/2 status - WT). The tumor characteristics of the other patient-derived cell lines may be found in previous reports [13, 14]. Notably, all patient-derived lines used in this study were authenticated as GBM by neuropathologists. Normal human astrocytes (Lonza: CC-2565) were cultured in AGM astrocyte growth medium (Lonza: CC-3187) supplemented with SingleQuots Supplements (CC-4123). All cells were cultured at 37 °C under a gas phase of 95% air and 5% CO_2_ and were tested for mycoplasma contamination (ATCC: 30-1012K) periodically throughout the study period. All studies were conducted within 10 passages and were authenticated using STR profiles.

### Antibodies, compounds, and plasmids

Primary antibodies AKT (4691), AKT-pS473 (4060), LAMP1 (9091S), LAMP1 (15665T), SQSTM1/p62 (88588S), SQSTM1/p62 (5114S), EGFR-pY1068 (2236S), EGFR (4267), CD71/TfR (13113), Synaptophysin (36406), LC3A/B (12741), LC3A/B (83506), PP2A-C (2038), PP2A-A (2041), PPP2R2A (5689), PPP2R5D (5687), and PP2A B Subunit (2290) were purchased from Cell Signaling Technologies. Antibodies targeting β-Tubulin (MA5-16308), PPP2R2B (PA5-29262), PPP2R5A (12675-2-AP), PPP2R5B (PA5-57740), PPP2R5C (39-3600), DRD2 (55084-1-AP), and PI(3,4,5)P_3_ (A-21328) were purchased from Thermo Fisher Scientific. PPP2R2D (PA5-30763) was obtained from Invitrogen. Antibodies targeting PPP2R5E (sc-515676) and SV40 ST (sc-58665) were obtained from Santa Cruz. Anti-PI(4,5)P_2_ (Z-P045) was purchased from Echelon Biosciences. PP2A-C-meL309 (ab66597) and PPP2R2C (ab27269) were purchased from abcam. PPP2R3C (MBS2523402) was obtained from MyBioSource. Secondary antibodies anti-rabbit IgG, HRP-linked (7074) and anti-mouse IgG, HRP-linked (7075) were purchased from Cell Signaling Technologies. Pimozide (P1793), Haloperidol (H1512), Perphenazine (P6402), Fluphenazine (F4765), Thioridazine (T9025), Chlorpromazine (C8138), Asenapine (A7861), Amisulpride (A2729), Quetiapine (Q3638), Paliperidone (P0099), Risperidone (R3030), Olanzapine (O1141), Iloperidone (SML1528), Clozapine (C6305), Lurasidone (L-030), Ziprasidone (1724408), Aripiprazole (SML0935), Fluoxetine (F132), Sertraline (S6319), Escitalopram (E4786), Citalopram (Y0001007), Duloxetine (D-044), Amitriptyline (A8404), Cyclobenzaprine (C-060), Raclopride (R121), SKF-38393 hydrochloride (D047), SCH-23390 (D054), Bafilomycin A1 (19-148), Quinpirole (Q102), Necrostatin-1 (480065), Z-VAD-FMK (V116), TNF-α recombinant protein (GF314), Staurosporine (19-123), and Erastin (329600) were purchased from Sigma. Ferrostastin-1 (S7243) was obtained from Selleckchem. Dopamine (A11136.22) was purchased from Thermo.

### Plasmids

LentiCRISPR_V2 (98290), SV40 ST (37858), ATG4B-C74A (21076), and the myc-BioID2-13x Linker-MCS plasmid (92308) were obtained from Addgene.

### Proximity ligation assay

Cells were seeded, fixed, permeabilized and blocked as outlined in the immunofluorescence method. Proximity ligation assay was performed using the Duolink in Situ Detection Reagents Red (Sigma: DUO92008) according to manufacturer’s instruction. Rabbit and mouse antibodies targeting our proteins of interest were added and incubated overnight at 4 °C on a rocker. Anti-mouse Plus probe (Sigma: DUO92001) and anti-rabbit Minus probe (Sigma: DUO92005) were added to the samples for 1 h in a humidified chamber at 37 °C. The ligase was added for 30 min followed by incubation with the polymerase for 100 min at 37 °C. Amplification was quenched via multiple washes before slides were mounted and sealed. Images were acquired using the Cytation 5 imager (Biotek).

### LysoTracker

Approximately 1.5 × 10^4^ cells were seed per well in an 8-well chamber slide and incubated for 24 h. 1:100 Hoechst 33342 (Thermo: R37605) was added at seeding. Cells were then treated with vehicle or drug for 1 h. Lysotracker deep red (Thermo: L12492) was added to the cells to a final concentration of 50 nM. Cells were incubated for 30 min at 37 °C. Cells were washed 2 times with media and images were acquired using the Cytation 5 instrument (Biotek). All images were captured within 15 min of Lysotracker removal to avoid loss of fluorescent signal.

### Acid sphingomyelinase activity assay

Acid sphingomyelinase activity was measured using the acid sphingomyelinase activity assay kit (Echelon: K-3200) according to the manufacturers’ protocol. Cells were lysed in RIPA buffer (Thermo: J63306.AP) containing protease and phosphatase inhibitors (Thermo: 78440). Lysates were subjected to 3 freeze-thaw cycles with 30 s of vortexing between cycles, followed by 30 min on a rotator at 4 °C. Protein quantification was carried out using the Pierce Rapid Gold BCA Protein Assay Kit (Thermo: A53225). 40 μg protein was aliquoted from each sample and lyophilized using a speedvac (10 h, room temperature). Samples were resuspending in sample buffer at a concentration of 10 μg/50 μL. 50 μL were added to a 96-well plate in triplicate along with standard. aSMase substrate was added to all samples and standards and the plate was incubated for 3 h at 37 °C on a rocker. Stop solution was added to all wells, followed by incubation at room temperature for 30 min. Fluorescence was measured using the Cytation 5 imager (Biotek).

### Western blotting

Cells were lysed in RIPA buffer (Thermo: J63306.AP) containing protease and phosphatase inhibitors (Thermo: 78440). Lysates were thoroughly mixed for 30 min on a rotator at 4 °C. Lysates were then sonicated (Branson 450 Digital Sonifier) ten times for 1 s at a 10% amplitude and cleared via centrifugation at 12000 × *g* and 4 °C for 20 min. Protein levels were determined and loaded onto 10% SDS-PAGE gels and transferred onto 0.2 μM PVDF membranes. Membranes were blocked with 5% BSA for 1 h at room temperature and probed with primary antibodies in 5% BSA overnight at 4 °C. Membranes were washed three times in TBST for 5 min and probed with the appropriate horseradish peroxidase-labeled secondary antibody for 1 h at room temperature. Signals were detected using Immobilon Western Chemiluminescent HRP Substrate (Millipore: WBKLS0500).

### Immuno-affinity purification of vesicles

Antibodies were conjugated to Dynabeads M-270 Epoxy magnetic beads at a concentration of 5 μg antibody per mg beads using the Dynabeads Antibody Coupling Kit (Thermo: 14311D). 5 mg beads were used per assay. Approximately 2 × 10^6^ cells were seeded in T75 flasks and incubated overnight. Vehicle or drug was added to the cells for 24 h. Cells were trypsinized and centrifuged. 10% of cells were aliquoted to quantify protein, lysates were normalized accordingly. 90% of cells were used for vesicle immuno-affinity precipitation. We used the Lysosome Enrichment Kit (Thermo: 89839) to gently lyse cells without disrupting vesicle membrane integrity, according to the manufacturer’s protocol. Cell plasma membrane was disrupted using 11 bursts of sonication (Branson 450 Digital Sonifier) for 1 s at a 10% amplitude. Plasma membrane degradation was confirmed by aliquoting 5 μL lysates onto coverslips and monitoring by microscopy. Lysates were centrifuged to clear heavy membranes. Supernatant was extracted and added to antibody conjugated beads. Samples were incubated for 4 h at 4 °C on a rotator. Magnetic beads were then isolated using a magnet and washed four times with PBS. 20% of samples were aliquoted for protein isolation. RIPA containing 1x lamelli buffer and β-me was added directly to samples. Samples were boiled, centrifuged and beads were extracted using a magnetic. 80% of samples were processed for mass spectrometry analysis. Vesicles were resuspended in acetonitrile and water (2:1), followed by 3 freeze-thaw cycles to shear membranes. Samples were vortexed for 30 s between cycles. Beads were removed from samples using a magnet. Samples were then filtered through 0.2 μm syringe filters (Agilent: 5190-5094) to remove contaminants.

### Small molecule steric hinderance score

The steric hinderance score of the most basic nitrogen was calculated according to the formulation developed by Kornhuber et al. [15]. Briefly, the largest substituent (determined by heavy atom count) at the most basic nitrogen atom is not considered because it is assumed to bind and partition into the inner leaflet of the lysosomal membrane. The heavy atom count (all atoms expect hydrogen) of the other two substituents is added up to determine the *k* steric hinderance score. If the most basic nitrogen is in a single ring not containing a double bond, then the neighbor atoms are cleaved, nitrogen atom deleted, and the heavy atom count of the smaller fragment determines the steric hinderance score. If the most basic nitrogen is common to two or three annulated rings or in a single ring with a double bond a *k* value of 6 is assigned. This penalty is related to the poor steric accessibility of the nitrogen atom (case A) or mesomeric delocalization of the positive charge (case B).

### Statistical analysis

**Fig. 1:**
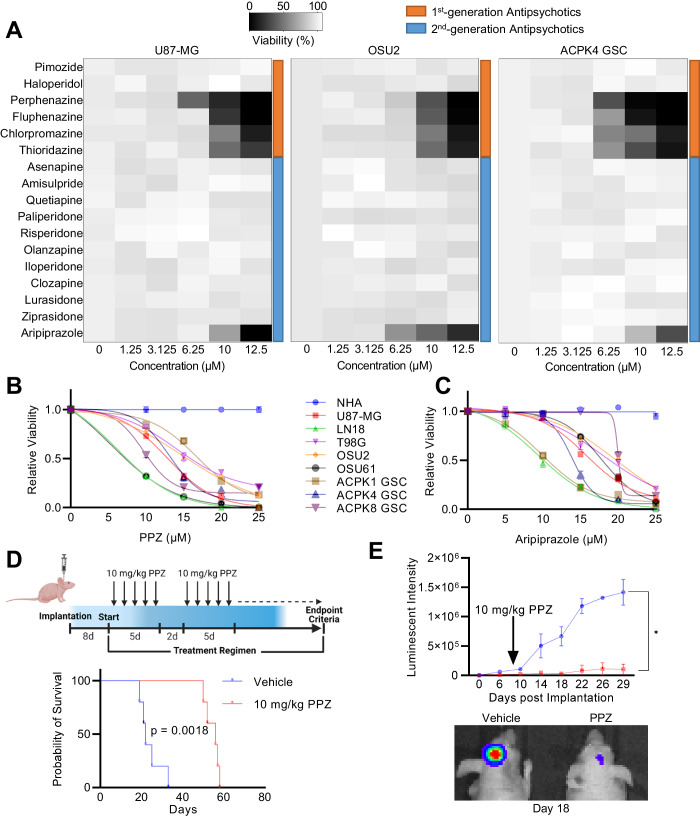
Identification of first- and second-generation antipsychotics with anti-GBM properties. **A** Heatmaps representing U87-MG, OSU2, and ACPK4 GSC cell viability 48 h after treatment with 1st- and 2nd generation antipsychotics. **B** Relative viability of NHAs, commercially available and patient-derived GBM cell lines 24 h following perphenazine treatment. **C** Relative viability of NHAs, commercially available and patient-derived GBM cell lines 24 h following aripiprazole treatment. **D** Kaplan–Meier curves of mice intracranially implanted with 100,000 U87-MG-Luc2 cells and treated with vehicle or 10 mg/kg perphenazine. Treatments started 8 days post-implantation and were administered 5 consecutive days followed by a two-day break, until endpoint criteria was met. **E** Bioluminescence quantification of U87-MG-Luc2 derived tumors in mice treated with vehicle or perphenazine. Representative images of mice treated with vehicle or perphenazine 18 days post implantation.

**Fig. 2:**
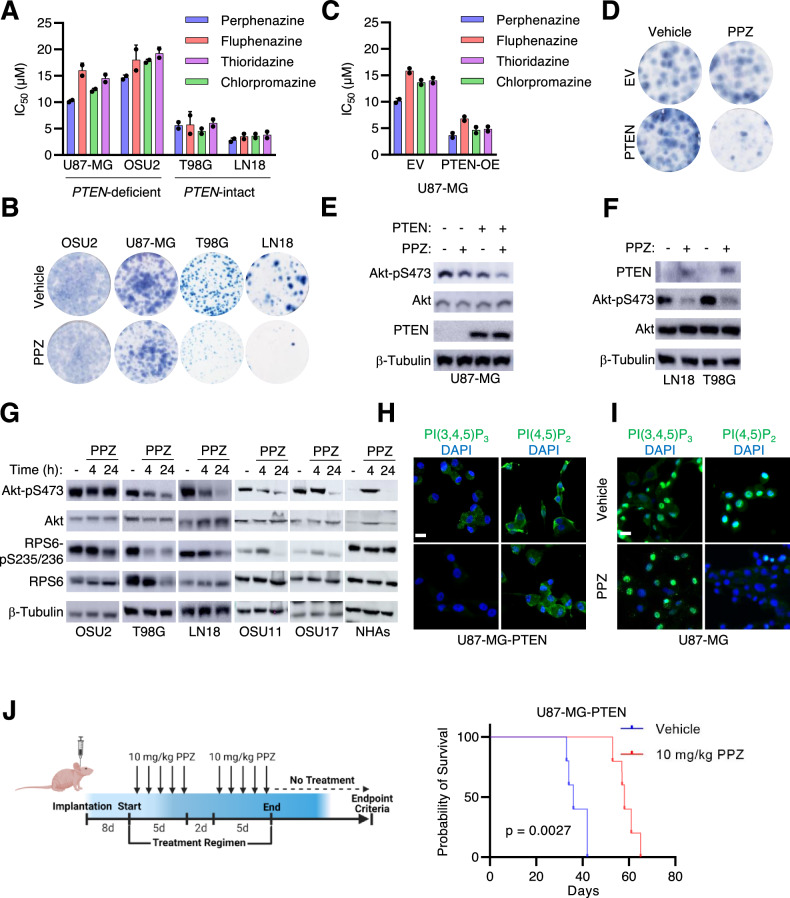
*PTEN* status dictates sensitivity to perphenazine. **A** IC_50_ values of phenothiazine compounds in the indicated cell lines after 48 h of treatment. **B** Representative clonogenic images of 200 cells 14 days post treatment with vehicle of 1.2 μM perphenazine. **C** IC_50_ of phenothiazines 48 h after drug treatment. **D** Representative clonogenic images of 200 cells 14 days post treatment with vehicle of 1.2 μM perphenazine. **E** Western blot analysis of empty vector (EV) and PTEN overexpression U87-MG cells treated with either vehicle or 10 µM perphenazine for 24 h. **F** Western blot analysis of T98G and LN18 cells treated with either vehicle or 10 µM perphenazine for 48 h. **G** Western blot analysis of Akt and RPS6 activation at 4 and 24 h after treatment with 10 µM perphenazine. **H**, **I** Images showing PI(3,4,5)P_3_ and PI(4,5)P_2_ levels in U87-MG-PTEN and U87-MG cells treated with either vehicle or 10 µM perphenazine for 48 h. **J** Kaplan-Meier survival curves of mice intracranially implanted with 100,000 U87-MG-PTEN cells and treated with either vehicle or 10 mg/kg perphenazine. A total of ten treatments were administered starting on day 8 post-implantation. Treatments were given for five consecutive days followed by a 2-day break for 2 weeks.

**Fig. 3:**
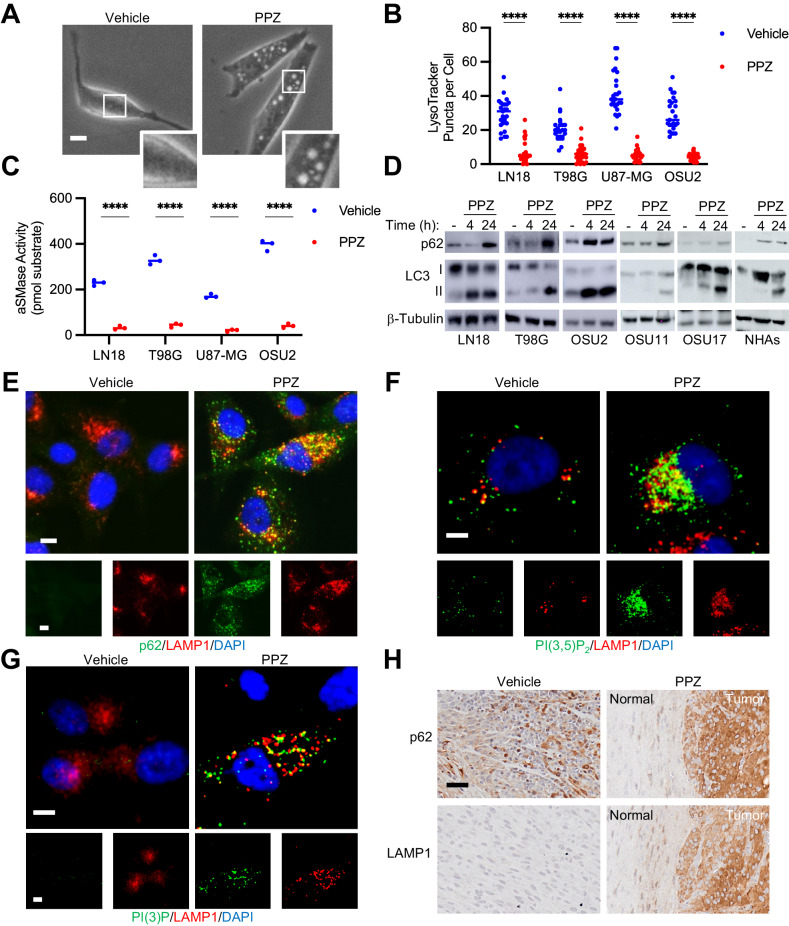
Perphenazine disrupts lysosomal function, hindering autophagic and endocytic flux. **A** Phase contrast images of U87-MG cell treated with vehicle or 10 μM perphenazine for 24 h. Scale bar: 5 μm. **B** Quantification of lysotracker puncta 24 h following treatment with vehicle of 10 μM perphenazine. **C** aSMase activity 24 h after treatment with vehicle or 10 μM perphenazine. **D** Assessment of indicated protein levels using western blot 4 and 24 h after treatment with 10 μM perphenazine. **E** Representative images of p62 and LAMP1 co-localization using immunofluorescent staining. Scale bar: 10 μm. **F** Representative images of PI(3,5)P_2_ and LAMP1 co-localization using immunofluorescent staining. Scale bar: 5 μm. **G** Representative images of PI(3)P and LAMP1 co-localization using immunofluorescent staining. Scale bar: 5 μm. **H** LAMP1 and p62 immunohistochemical staining of U87-MG tumors treated with vehicle or perphenazine.

**Fig. 4:**
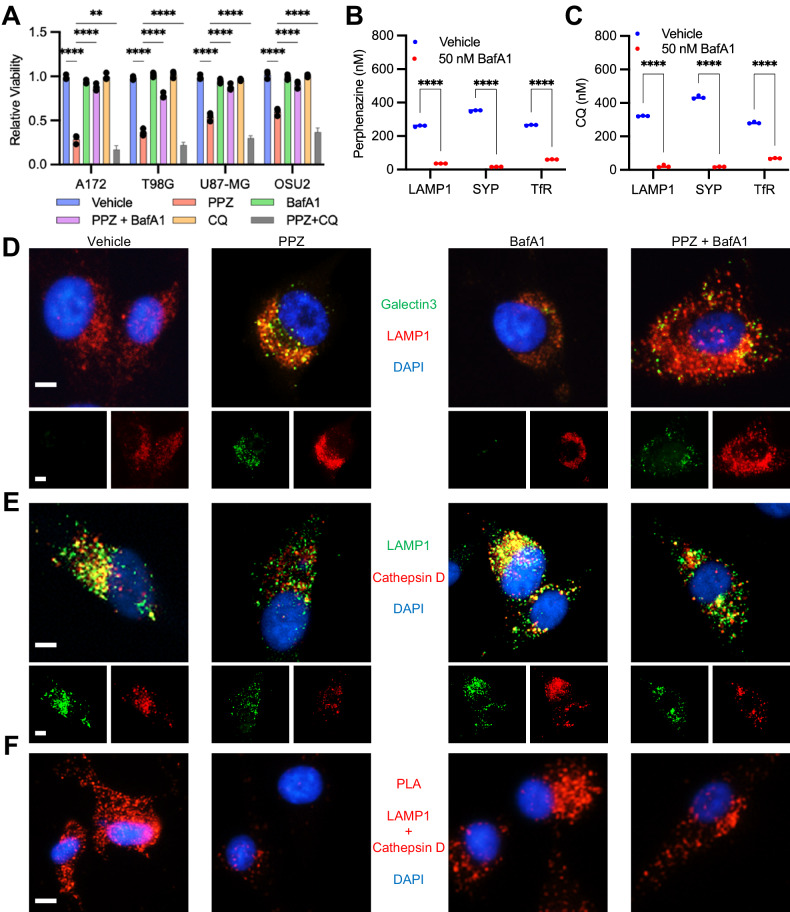
Perphenazine accumulates in lysosomes triggering lysosomal membrane permeability and cell death. **A** Relative cell viability 24 h following treatment with vehicle, 50 nM BafA1, 50 μM CQ, and/or 15 μM perphenazine. BafA1 and CQ were added 1 h prior to the addition of perphenazine. **B** LC-MS/MS quantification of perphenazine in isolated vesicles 24 h after treatment with vehicle or 50 nM BafA1. **C** LC-MS/MS quantification of CQ in isolated vesicles 24 h after treatment with vehicle or 50 nM BafA1. **D** Representative images of Galectin 3 and LAMP1 co-localization 24 h after treatment with vehicle, 15 μM perphenazine and/or 50 nM BafA1 in U87-MG cells. Scale bar: 5 μm. **E** Representative images of Cathepsin D and LAMP1 24 h after treatment with vehicle, 15 μM perphenazine and/or 50 nM BafA1 in U87-MG cells. Scale bar: 5 μm. **F** Proximity ligation assays measuring the co-localization of LAMP1 and Cathepsin D 24 h after treatment with vehicle, 15 μM perphenazine and/or 50 nM BafA1 in U87-MG cells. Scale bar: 5 μm.

**Fig. 5:**
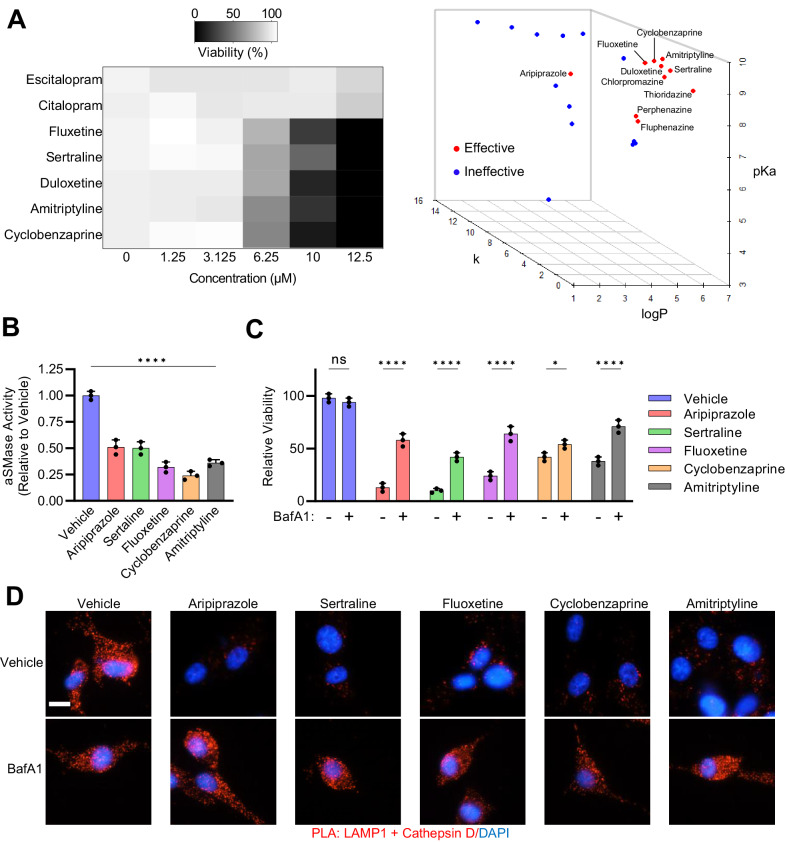
Chemical properties predict antipsychotic cytotoxicity in GBM. **A** Left: Viability of U87-MG cells 48 h following treatment with the indicated drugs. Right: 3-D plot mapping the effective and ineffective compounds by their chemical attributes. *k* = steric hinderance score. **B** aSMase activity assays following treatment with the indicated drugs (10 μM) for 24 h. **C** Viability of U87-MG cells 48 h following 10 μM treatment with the indicated drugs ±a 1 h pre-treatment with vehicle or 50 nM BafA1. **D** Proximity ligation assays measuring the co-localization of LAMP1 and Cathepsin D in U87-MG cells, 24 h after the indicated treatments. Cells were treated with vehicle, 10 μM antipsychotic and/or 50 nM bafilomycin A1. Bafilomycin A1 was added 1 h prior to treatment with antipsychotics.

**Fig. 6:**
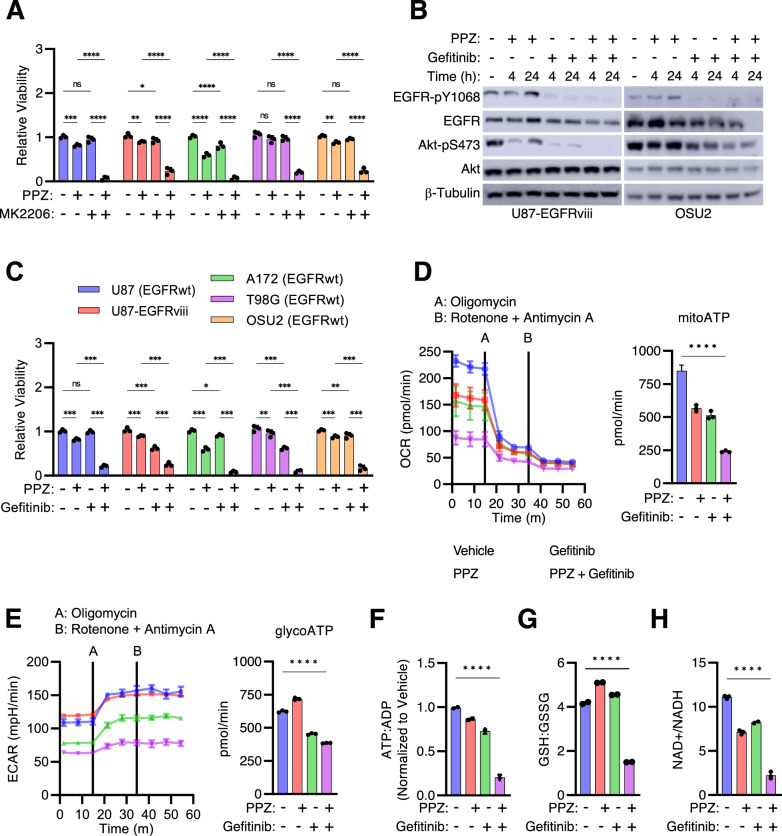
EGFR-PI3K-Akt pathway inhibition increases perphenazine sensitivity. **A** Cell viability 24 h after treatment with vehicle, 10 μM perphenazine and/or 10 μM Gefitinib. **B** Western blots measuring EGFR and Akt activation 24 h after 10 μM perphenazine and/or 10 μM Gefitinib treatment. **C** Cell viability 24 h after treatment with vehicle, 10 μM perphenazine and/or 2.5 μM MK-2206. **D**, **E** Real-time ATP production from glycolysis and OXPHOS was determined using the Seahorse XF Real-Time ATP Rate Assay. Cells were treated with the indicated drugs for 1 h prior to starting the assay. ATP production was normalized to cell count. **F** LC-MS/MS quantification of the ATP/ADP ratio 24 h following the indicated treatments. **G** LC-MS/MS quantification of the GSH/GSSG ratio 24 h following the indicated treatments. **H** NAD^+^/NADH ratio was quantified 24 h following the indicated treatments.

Statistical methods were not used to predetermine sample size. Data are presented as a mean of individual biological experiments or technical replicates as indicated in figure legends. Error bars represent standard deviation. Unpaired t-tests were used to calculate *p* values for Figs. 1E, 3B, C, 4A–C, 5B, C, 6A, C–H, S1B, S1D, S2A–C, S3F–H, and S5A–G. Log-rank test was used to calculate *p* values of Kaplan-Meier survival analysis in Figs. 1D and 2J. All statistical testing was conducted in GraphPad Prism. *p* < 0.05 was considered statistically significant. All center values represent mean values, and all error bars represent standard deviation.

For xenograft studies, a sample size 5 mice per treatment group was used for all xenograft studies. No randomization or blinding of animals was performed. However, we ensured tumor size was similar across animals prior to treatment with vehicle and perphenazine. The sample size for xenograft studies was determined based on the power calculation from an in vivo experiment performed previously. It was determined that 5 mice would be sufficient to determine an effect size of 5 days with a power of 0.90 and a significance level of 0.05.

### Supplemental methods

Information on small interfering RNA, intracranial xenografts, Bioluminescence imaging, MTS viability assay, Immunofluorescence, CRISPR-Cas9, SV40 ST transfection, NAD^+^/NADH quantification, Seahorse XF real-time ATP rate assay kit, and Triple quad LC-MS/MS methods are provided as supplemental information. Ion mode, retention time, and quantifier/qualifier ions used for MRM quantification of metabolites are in Supplemental Fig. S6C, D.

## Results

### Identification of first- and second-generation antipsychotics with anti-GBM properties

Cytotoxicity screens were conducted in three different GBM cell lines: commercially available U87-MG, patient-derived adherent OSU2, and low passage patient-derived ACPK4 cells grown in serum free conditions. Our results demonstrated that the first-generation phenothiazines (perphenazine, fluphenazine, chlorpromazine, and thioridazine) and the second-generation aripiprazole exhibited similar cytotoxicity across the three cell lines (Fig. 1A). We confirmed the cytotoxic effect of perphenazine and aripiprazole across commercially available and patient-derived GBM cell lines as well as normal human astrocytes (NHAs) (Fig. 1B, C). NHAs were unaffected by either drug at concentrations up to 25 μM, whereas all GBM cells showed sensitivity at concentrations between 5-15 μM, suggesting a therapeutic index. Perphenazine demonstrated the most potent effect and was selected for further investigation.

We next assessed the therapeutic potential of perphenazine in vivo by monitoring its influence on tumor growth, survival, and neurological symptoms in nude mice intracranially implanted with U87-MG-Luc2 cells. Starting eight days after implantation, mice received 10 mg/kg perphenazine treatment for five consecutive days, followed by a two-day break, until they met endpoint criteria. Compared to mice treated with vehicle (25 ± 5 days), perphenazine treatment doubled mouse survival (55 ± 4 days) and significantly reduced tumor growth (Fig. 1D, E), revealing potent anti-GBM activity under in vivo conditions. These results provide evidence that perphenazine may be a promising candidate to treat GBMs.

### *PTEN* status dictates sensitivity to perphenazine

In our cytotoxicity assays, we observed a relationship between the sensitivity of GBM cells to antipsychotic drugs and the status of *PTEN*, a tumor suppressor gene that negatively regulates the PI3K-Akt signaling pathway. *PTEN* intact GBM cell lines exhibited enhanced sensitivity to the first antipsychotics possessing anti-GBM activity (Fig. 2A, B). To directly assess the influence of PTEN expression on antipsychotic sensitivity, we utilized isogenic U87-MG PTEN overexpression cells. The forced expression of PTEN increased sensitivity across antipsychotics (Fig. 2C: Supplemental Fig. S1A). This corresponded with a reduction in colony formation following perphenazine treatment compared to parental cells (Fig. 2D). In U87-MG-PTEN cells, perphenazine decreased the activation of Akt (Fig. 2E). Intriguingly, this corresponded with an increase in the total levels of PTEN (Fig. 2E). Similar findings were observed in *PTEN* intact GBM cell lines, where perphenazine treatment also increased PTEN levels, leading to inhibition of oncogenic signaling nodes Akt/mTORC1 (Fig. 2F, G). Notably, in NHAs, perphenazine treatment transiently increased Akt activation while RPS6 activation remained unchanged, suggesting the influence of perphenazine on oncogenic signaling may be cancer cell specific. PTEN controls PI3K-Akt activity by dephosphorylating the phosphoinositide PI(3,4,5)P_3_ to PI(4,5)P_2_. To assess the functional impact of perphenazine-induced PTEN stabilization, we conducted immunofluorescence assays to measure changes in PI(3,4,5)P_3_ and PI(4,5)P_2_. We observed that perphenazine treatment decreased PI(3,4,5)P_3_ and increased PI(4,5)P_2_ in *PTEN* intact T98G cells and U87-MG-PTEN cells (Fig. 2H; Supplemental Fig. S1B). In contrast, *PTEN* deficient U87-MG cells showed no change in PI(3,4,5)P_3_ levels and a decrease in PI(4,5)P_2_ following perphenazine treatment (Fig. 2I). These findings suggest that perphenazine enhances phosphatase activity by stabilizing PTEN, which in turn depletes PI(3,4,5)P_3_ and leads to the suppression of Akt activity.

We next assessed whether *PTEN* status influenced the anti-GBM activity of perphenazine in vivo. Short-term and long-term assays demonstrated that U87-MG-PTEN cells were ~3 times more sensitive to perphenazine as parental U87-MG cells (Fig. 2C, D). To assess whether this enhanced sensitivity was maintained under in vivo conditions, we decided to administer 10 total doses of 10 mg/kg perphenazine to mice bearing U87-MG-PTEN cells, corresponding to ~1/3 of the average 34 total doses administered to mice implanted with U87-MG cells. Treatment was initiated 8 days post-implantation and consisted of a 5-day course of perphenazine followed by a two-day rest period. Perphenazine treatment led to a significant survival advantage (59 ± 6 days) compared to vehicle-treated controls (35 ± 5 days) (Fig. 2J). This established *PTEN* status as a key determinant of perphenazine sensitivity in GBM.

### Cytotoxic effect of perphenazine is independent of dopamine receptor antagonism and PP2A activation

We next investigated perphenazine’s cytotoxic mechanism in GBM, initially exploring its role as an antagonist of dopamine receptors 1 and 2 (DRD1/2) [16, 17]. We found no DRD1 in GBM cell lines, whereas T98G and LN18 expressed DRD2 (Supplemental Fig. S1C). However, perphenazine-induced cell death remained unaffected by pharmacologic modulation of DRD2 or siRNA targeting DRD2 in T98G cells (Supplemental Fig. S1D–F). We next assessed whether perphenazine-induced cell death resulted from the inhibition of an off-target protein. Drug target predictions from the Cancer Dependency Map (https://depmap.org/portal/) suggested that perphenazine possessed affinity for additional neurotransmitter receptors (HRH1, HTR2A, HTR2C, HTR6, HTR7). However, CRISPR-Cas9 and shRNA library screens provided in the Cancer Dependency Map demonstrated no dependency of any neurotransmitter receptor genes across GBM cell lines (Supplemental Fig. S1G, H).

We then explored the possibility that perphenazine’s cytotoxicity is linked to its PP2A-activating properties [18]. Inhibiting PP2A using LB-100, siRNAs targeting the PP2A catalytic subunit, or overexpressing SV40 ST antigen did not rescue perphenazine-induced cell death (Supplemental Fig. S2A-C). We hypothesized that perphenazine might alter specific PP2A trimeric complexes. Upon transfection of myc-BioID2-PP2A-Aα fusion protein, we observed changes in PP2A trimer composition, including decreased B55α and increased B56γ, B56δ, and B56ε subunits (Supplementary Fig. S2D). Total levels of these PP2A-B subunits did not change following perphenazine treatment, suggesting this was not a result of changes in expression level (Supplementary Fig. S2E). CRISPR-Cas9 knockout of these subunits did not reverse perphenazine’s cytotoxicity in U87-MG cells (Supplemental Fig. S2F, G). Thus, we concluded that perphenazine’s cytotoxic effect in GBM is likely independent of both dopamine receptor antagonism and PP2A activation.

### Perphenazine disrupts lysosomal function, hindering autophagic, and endocytic flux

We next delved into alternate mechanisms of cytotoxicity of perphenazine. We started with a microscopic observation that perphenazine treatment induces the formation of cytoplasmic vacuoles in GBM cells (Fig. 3A). We noticed that the morphological changes were akin to various forms of lysosomal disruption, including lysosomal storage disorders, as well as the isogenic blockage of lysosomal function [19, 20]. Similar to several lysosomal storage disorders, perphenazine treatment resulted in the accumulation of lipid-filled perinuclear vesicles (Supplementary Fig. S3A). We therefore investigated the influence of perphenazine on lysosomal function. Lysosomes, which are acidic organelles that act as the digestive system of the cell, are responsible for degrading cargo from both the autophagic and endocytic vesicle trafficking pathways. Impairment of lysosomal function is crucial for the growth and progression of diverse cancer types [21–23]. In this context, lysosome disruption is emerging as a potential strategy to hinder tumor cell growth, owing to the preferential sensitivity of cancer cells to lysosome-altering agents [24]. We therefore posited that the cytotoxic effect of perphenazine might be linked to the inhibition of lysosomal function. To test lysosomal function directly, we measured lysosomal acidity and enzymatic activity following perphenazine treatment. Lysosomal acidity (pH 4–5) is necessary for the optimal activity of catabolic enzymes, making it a surrogate for lysosomal activity. Perphenazine decreased the puncta formed by lysotracker dye, which accumulates specifically in acidic lysosomes, demonstrating that perphenazine reduced active lysosomes (Fig. 3B; Supplementary Fig. S3B). Further, perphenazine treatment decreased the activity of the pH-sensitive lysosomal enzyme acid sphingomyelinase (aSMase) (Fig. 3C), supporting the notion that perphenazine inhibits lysosomal activity in GBM.

To validate the disruption of lysosomal function, we measured perphenazine-induced changes in both autophagic and endocytic pathways. We evaluated macroautophagy, referred to as autophagy within this study, by analyzing changes in two key indicators of autophagic activity: LC3 lipidation (LC3 II) and p62 [25]. When autophagy is triggered, LC3 undergoes cleavage and lipidation, resulting in the formation of LC3 II, which is subsequently recruited to autophagosomal membranes. However, inhibiting autophagic flux also leads to elevated LC3 II levels due to hindered degradation processes. To discern whether heightened LC3 II levels signify the initiation of autophagy or its obstruction, we concurrently quantified the levels of p62. Serving as an autophagy substrate, p62 is normally broken down by lysosomes during autophagy. Conversely, when autophagic flux is impeded, p62 accumulates. Perphenazine induced an increase in both LC3 II and p62 levels across GBM cell lines (Fig. 3D). This observation was consistent in NHAs, suggesting that perphenazine may also possess autophagy-disrupting properties in normal cells. This indicated an initiation of autophagy (increased LC3 II levels) but a decrease in lysosomal degradation of autophagic cargo (increase in p62 levels), supporting a blockage of lysosomal function. To affirm this disruption of autophagic flux, we assessed changes in these autophagy markers by perphenazine under autophagy-inducing conditions. Perphenazine maintained the inhibition of autophagic flux following glucose, glutamine, and serum deprivation, resulting in significant growth suppression (Supplementary Fig. S3C–H). Decreased autophagic flux may result from a decrease in autophagosome-lysosome fusion or decreased catabolic activity of fused autophagosome-lysosome vesicles (autolysosomes). To evaluate these two possibilities, we conducted co-localization using markers of autophagosomes and lysosomes. Lysosomes were monitored using the lysosomal-associated membrane glycoprotein LAMP1 that plays a role in lysosomal integrity and catabolic activity. Perphenazine treatment increased the co-localization of p62 and LAMP1 (Fig. 3E), indicating that autophagic flux was blocked after autolysosome formation. This co-localization was confirmed using proximity ligation assays (Supplementary Fig. S3I).

To assess the impact of perphenazine on endocytic flux, we evaluated the intracellular accumulation of receptors regulated by endocytosis, including the transferrin receptor (TfR) and the epidermal growth factor receptor (EGFR). Perphenazine treatment induced an increased accumulation of these receptors in a time-dependent manner (Supplementary Fig. S4A). Immunofluorescent imaging demonstrated that the accumulation of these receptors was intracellular, consistent with a disruption in endocytic flux (Supplementary Fig. S4B). We next monitored the levels of phosphatidylinositols PI(3)P and PI(3,5)P_2_, lipids that play a critical role in endocytic trafficking and vesicle fusion. Our analysis revealed a marked increase in the accumulation of these endosomal markers in perphenazine-treated cells (Supplementary Fig. S4A–D). Further investigation showed that perphenazine induced the formation of endosome-lysosome fused vesicles (endolysosomes), as demonstrated by the co-localization of PI(3)P and PI(3,5)P_2_ with the lysosomal marker LAMP1 (Fig. 3F, G, Supplemental Fig. S4E, F). Finally, we demonstrated that perphenazine induced a significant increase in p62 and LAMP1 in tumor cells compared to normal surrounding tissues (Fig. 3H). Together these results demonstrate that perphenazine impairs lysosomal function, leading to an accumulation of autolysosomes and endolysosomes in GBM cells.

### Perphenazine accumulates in lysosomes triggering lysosomal membrane permeability and cell death

In cancer cells, lysosomal dysfunction can trigger various forms of cell death, including apoptosis, necroptosis, ferroptosis, and autophagy depending on the cellular context [26–29]. To elucidate the mechanism underlying the cytotoxicity of perphenazine, we sought to investigate the induction of these different forms of cell death. To this end, we pre-treated cells with specific inhibitors of necroptosis (necrostatin-1), apoptosis (Z-VAD-FMK), and ferroptosis (ferrostatin-1) and assessed their ability to rescue perphenazine-induced cytotoxicity. We observed that the pre-treatment of cells with these inhibitors failed to rescue perphenazine-induced cell death, despite their ability to reverse cell death induced by positive controls such as TNFα, Staurosporine, and Erastin, respectively (Supplementary Fig. S5A–F). Similarly, inhibition of autophagy using siRNAs targeting ATG5 and ATG7 as well as overexpression of the dominant-negative ATG4 (C74A) mutant did not rescue perphenazine-induced cell death (Supplemental Fig. S5G), suggesting cell death was independent of changes in autophagy. Finally, Annexin V / PI analysis confirmed that perphenazine induced cell death was independent of apoptosis and necrosis (Supplementary Fig. S5H).

Subsequently, we investigated whether perphenazine could induce lysosomal membrane permeabilization (LMP), an emerging cellular demise mechanism triggered by the cytotoxic buildup of lipids, reactive oxygen species, and/or small molecules within lysosomes. This culminates in the disintegration of the lysosomal membrane [15, 24, 30], resulting in the release of catabolic enzymes into the cytosol and subsequent cellular degradation. To assess this phenomenon, we pretreated cells with bafilomycin A1 (BafA1), an inhibitor of vacuolar-type ATPase (V-ATPase), prior to perphenazine treatment. BafA1 has been demonstrated to counteract the accumulation of lysosome-targeting compounds within lysosomes [31]. Our hypothesis posited that if perphenazine indeed induces LMP, BafA1 might mitigate this effect by preventing perphenazine from entering the lysosomal compartment. Efficaciously, pre-treatment with BafA1 rescued perphenazine-induced cytotoxicity across multiple glioblastoma cell lines (Fig. 4A).

We next employed liquid chromatography-tandem mass spectrometry (LC-MS/MS) to quantitatively assess perphenazine levels in immuno-affinity purified lysosomes (anti-LAMP1), endosomes (anti-TfR), and synaptic vesicles (anti-Synaptophysin) (Supplementary Fig. S6A). This analysis aimed to verify that BafA1 hindered the accumulation of perphenazine within V-ATPase-containing vesicles. Notably, while lysosomes and late endosomes rely on V-ATPases for maintaining the requisite pH for catabolic enzyme activity, synaptic vesicles utilize V-ATPases to establish the proton gradient necessary for neurotransmitter loading [32]. The LC-MS/MS data revealed an accumulation of perphenazine in all three types of vesicle isolations, a buildup effectively abrogated by BafA1 pretreatment (Fig. 4B).

Furthermore, our findings revealed that perphenazine exhibited a more pronounced inclination towards lysosomal disruption when contrasted with the extensively utilized lysosome-neutralizing agent, chloroquine (CQ). Notably, pretreatment with CQ did not heighten or ameliorate perphenazine-induced cytotoxicity (Fig. 4A), suggesting distinct mechanisms of lysosome disruption. Despite comparable levels of accumulation within vesicles (Fig. 4C), perphenazine induced cytotoxicity, while achieving a 2–3-fold higher concentration of CQ was necessary to achieve a similar reduction in GBM viability (Supplementary Fig. S6B). Hence, we inferred that perphenazine possesses a unique property of lysosomal disruption that diverges from the conventional lysosome-disrupting actions of CQ.

We proceeded to investigate whether perphenazine caused lysosomal membrane damage by performing co-localization experiments. Our goal was to directly measure LMP following perphenazine treatment. To assess whether perphenazine induced lysosomal membrane damage, we evaluated the co-localization of Galectin 3, a protein recruited to lysosomal membranes to initiate repair [33], and the lysosomal membrane protein LAMP1. The results showed that perphenazine treatment increased the co-localization of Galectin 3 and LAMP1, indicating that perphenazine caused lysosomal membrane damage (Fig. 4D). We subsequently monitored the co-localization of the lysosomal protein Cathepsin D and lysosomal membrane protein LAMP1. The results revealed that perphenazine treatment decreased Cathepsin D and LAMP1 co-localization, suggesting that the lysosomal membrane had broken down and released catabolic enzymes into the cytosol (Fig. 4E). We confirmed the decrease in Cathepsin D-LAMP1 co-localization using proximity ligation assays (Fig. 4F). Notably, pre-treatment with BafA1 effectively reversed the perphenazine-induced increase in Galectin 3-LAMP1 co-localization and decrease in Cathepsin D-LAMP1 co-localization (Fig. 4D–F), consistent with reversing perphenazine induced cell death. Therefore, we concluded that perphenazine exhibits membranolytic properties that induce LMP and cell death in GBM.

### Chemical properties predict antipsychotic cytotoxicity in GBM

There is a striking similarity in the chemical properties that dictate BBB permeability and lysosome accumulation, including logP, pKa, protonizability, and tertiary nitrogen atoms [34, 35]. Based on this observation, we predicted that other clinically used CNS drugs may also exhibit anti-GBM activity. We measured the cytotoxicity of a panel of new generation and commonly prescribed CNS drugs in GBM (Fig. 5A). We observed that drugs capable of effectively decreasing viability were highly lipophilic (logP > 4), weak bases (basic pKa 7.81–9.80), and possessed little steric hinderance (*k* < 4) (Fig. 5A; Supplementary Table 1). The steric hinderance calculation was conducted according to a previously developed model [15]. The exception was aripiprazole that demonstrated effectiveness despite possessing significant steric hindrance (*k* = 13), possibly resulting from a lower pKa compared to noneffective counterparts. These new-generation CNS drugs effectively decreased aSMase activity, consistent with a reduction in lysosomal function (Fig. 5B). Pre-treatment of GBM cells with BafA1 rescued cell death in all cases (Fig. 5C). Proximity ligation assays demonstrated that this panel of small molecules induced LMP, indicated by the decrease in Cathepsin D-LAMP1 co-localization that was effectively reversed by pre-treatment with BafA1 (Fig. 5D). We concluded that the anti-GBM properties of CNS drugs are related to the disruption of lysosomal homeostasis and induction of lysosomal cell death.

### EGFR-PI3K-Akt pathway inhibition increases perphenazine sensitivity

Our findings demonstrate that perphenazine effectively impairs lysosomal function in GBM cells, regardless of their *PTEN* status (Fig. 3B, C). Yet, GBMs with intact *PTEN* exhibited a heightened sensitivity to perphenazine, corresponding with the inhibition of Akt and RPS6 activation. To explore this phenomenon further, we investigated whether the change in Akt activity contributed to this enhanced sensitivity. Co-treatment with an allosteric Akt inhibitor (MK-2206) or an EGFR inhibitor (Gefitinib) significantly enhanced perphenazine sensitivity in GBM cell lines, suggesting that activation of the EGFR-PI3K-Akt pathway modulates perphenazine sensitivity in GBM (Fig. 6A, C).

To understand why the simultaneous inhibition of Akt and lysosomal function leads to synergistic cell death, we hypothesized that this approach may disrupt the metabolic homeostasis of GBMs. Previous reports have shown that the combined inhibition of anabolic growth factor signaling and lysosome-dependent catabolism can block tumor growth by disrupting nutrient, energy, and/or antioxidant homeostasis [36–38]. To test this hypothesis, we treated the U87-MG-EGFRviii cell line, which was the most resistant to perphenazine and gefitinib treatment as single agents, with a combination of gefitinib and perphenazine. Our results showed that this combination significantly decreased the ATP:ADP ratio by collapsing ATP production from glycolysis and oxidative phosphorylation (Fig. 6D–F). The loss of ATP production correlated with a decreased redox potential, as indicated by a reduction in the NAD^+^/NADH and GSH:GSSG ratios (Fig. 6G, H). Based on these results, we conclude that the inhibition of the EGFR-PI3K-Akt pathway promotes perphenazine sensitivity by disrupting energy and redox homeostasis in GBM.

## Discussion

In this study, we identified multiple medications used to treat CNS disorders that possess cytotoxicity in GBM. These drugs include first- and second-generation antipsychotics, selective serotonin uptake inhibitors, serotonin and norepinephrine reuptake inhibitors, and tricyclic antidepressants. Despite their distinct neuromodulatory functions, we discovered that they share a common mechanism of cytotoxicity in GBM involving the destabilization of lysosomal membranes. This commonality is likely due to their shared lipophilicity and weak base properties, enabling them to effectively cross the BBB and target the lysosomal compartment. The significance of lysosomal integrity in GBM is reinforced by the pivotal role played by enhanced lysosomal integrity in glioma stem cells, which contributes to resistance against radiation therapy [39]. The notion that antipsychotics directly disrupt the lysosomal membrane is supported by reports that phenothiazines interact with phospholipids and are capable of altering lipid bilayer structures [40–44]. Our findings are further supported by previous works demonstrating that phenothiazines impede cholesterol efflux from lysosomes in melanoma and that fluoxetine inhibits the function of the lysosomal enzyme aSMase in GBMs [45, 46]. Our discovery that these drugs disrupt lysosomal function provides a rationale for previous reports demonstrating that phenothiazines modulate autophagy and endocytosis [47–49]. In future studies, these chemical characteristics may be useful in predicting the anti-GBM activity of other potential repurposed compounds not covered in this study.

Moreover, this study provides crucial insight into the mechanism of action and determinants of cytotoxicity of several neuromodulators currently undergoing clinical trials as repurposing agents for various cancers. These drugs include Fluoxetine, Sertraline, Thioridazine, Chlorpromazine, and Fluphenazine. Our observation that *PTEN* status and Akt activation determine the cytotoxic efficacy of antipsychotics suggests these drugs may be used for personalized treatment based on *PTEN* status in the clinic. This observation is in correspondence with prior work demonstrating that the antipsychotic chlorpromazine blocks Akt activation [50]. The enhanced sensitivity of *PTEN* intact GBMs to perphenazine corresponded with the stabilization of *PTEN* levels. In neurons, *PTEN* levels are regulated by lysosomal degradation in addition to ubiquitination [51], suggesting that perphenazine-induced inhibition of lysosomal function may increase PTEN levels. Inhibitors of the EGFR-PI3K-Akt pathway also synergistically sensitized *PTEN*-null or mutant GBMs to perphenazine, decreasing ATP levels and collapsing the redox and antioxidant potential of GBM cells. Therefore, future investigations could explore the possibility of augmenting the clinical efficacy of these therapies by targeting cancers with intact *PTEN* or in combination with inhibitors of the EGFR-PI3K-Akt pathway.

The differential susceptibility of GBMs and normal human astrocytes to perphenazine aligns with previous work indicating that lysosome-disrupting agents exhibit cancer-specific cytotoxicity [24]. Several pieces of evidence may explain the heightened sensitivity of cancer cells to lysosome-disrupting agents. Lysosomes within cancer cells are larger than lysosomes in non-transformed cells and exhibit diminished activity of lysosomal enzymes that stabilize membrane integrity, potentially rendering them more vulnerable to lysis [24, 52]. Cancer cells possess increased storage and turnover of iron, predominantly sequestered in lysosomes, amplifying the likelihood of LMP resulting from reactive iron species [53]. Finally, differences in the composition of phospholipid bilayers in normal and cancer cells [54] may make cancer cells more susceptible to lipid bilayer rupture following integration of LMP-inducing agents.

The potent anti-GBM properties of perphenazine observed in vivo may result from the disruption of synaptic signaling between GBM and normal tissues in addition to disrupting lysosomal integrity. There is emerging evidence that synaptic connections between GBM cells and neurons leads to an electrical integration that promotes glioma progression [55, 56]. Neuron-glioma circuits rely on vesicle trafficking that may be disrupted by perphenazine, evidenced by the accumulation of perphenazine in synaptic vesicles. It is therefore possible that the strong anti-GBM properties of perphenazine observed in our xenograft models is a result of, at least in part, a disruption in these electrical circuits demonstrated to drive GBM aggressiveness. Further, neuron-GBM synaptic signaling is reported to be particularly important for breast-to-brain metastasis [57]. This suggests that antipsychotics, like perphenazine, may disrupt breast cell integration into the brain, warranting further investigation.

DRD2 antagonism by phenothiazines is postulated to account for their ability to combat schizophrenia [58]. However, the assumption that dopamine receptor inhibition is responsible for their antipsychotic function (“dopamine hypothesis of schizophrenia”) remains controversial [59]. It is noteworthy that antipsychotic drugs physically impede lysosomal function in a V-ATPase-dependent manner. Given that synaptic vesicles rely on V-ATPases to generate the proton gradient necessary for neurotransmitter loading, it is conceivable that these compounds may similarly disrupt synaptic vesicle function. It is therefore intriguing to speculate that the accumulation of antipsychotic drugs within synaptic vesicles may disrupt abnormal neurotransmitter signaling under psychiatric conditions, contributing to the mechanism of action of effective neuromodulating medications.

### Supplementary information

Supplementary Information

Supplemental Figure 1 to Supplemental Figure 6

Supplemental Table

Raw western blot images

## Data Availability

The authors confirm that the data supporting the findings of this study are available within the article and/or its supplemental materials.
