# Supplementary material for: Antipsychotics possess anti-glioblastoma activity by disrupting lysosomal function and inhibiting oncogenic signaling by stabilizing PTEN
Source: Cell Death Dis. 2024 Jun 13;15(6):414. doi: 10.1038/s41419-024-06779-3 (PMC11176297; doi:10.1038/s41419-024-06779-3)
Supplement: Supplementary file 1 — Supplementary Information [file 41419_2024_6779_MOESM1_ESM.pdf]

Supplementary Materials

Supplemental Methods:

Small Interfering RNA

About $1x10^5$ cells per well were seeded in a 6-well plate. 24 hours after seeding, cells were treated with 100 nM small interfering RNA (siRNAs) mixed with 10 μL MISSION siRNA Transfection Reagent (Sigma: S1452). The following siRNAs (Sigma: 4390824) were used: Negative control (Sigma: SIC001), PPP2CA #1 (Sigma: s10957), PPP2CA #2 (Sigma: s10959), DRD2 #1 (Sigma: SASI_Hs02_00303242), DRD2 #2 (Sigma: SASI_Hs01_00092054), ATG5 (Sigma: SASI_Hs01_00173156), and ATG7 (Sigma: SASI_Hs01_00077649).

SV40 ST Transfection

Approximately $1x10^4$ cells were transfected with 1 mg of the SV40 ST plasmid (Addgene: 37858), using Lipofectamine 3000 (2 mL P3000, 2 mL Lipofectamine 3000) (Thermo: L3000001).

CRISPR-Cas9

LentiCRISPR_V2 (Addgene: 98290) was digested with BsmBI (NEB: R0739S) in NEB Buffer 3.1. Zymoclean large fragment DNA recovery kit (Zymo: D4045) was used to purify linear plasmid. Forward and reverse oligos were annealed in 1X NEB Buffer 3.1 and heat, forming overhangs that correspond with BsmBI overhangs. Subcloning of the guideRNA oligos and LentiCRISPR_V2 plasmid was carried out using electroLigase (NEB: M0369). The oligos used to generate each guideRNA are listed in the supplemental material. Cells were transfected at a MOI of approximately 10. Stable cells were established using

puromycin selection for 7 days. The following guideRNAs were deigned to target luciferase (control) and PP2A subunits.

Luciferase_1 (F/R):

CACCGTCACCAAGGAGCTGGACCAG\AAACCTGGTCCAGCTCCTTGGTGAC

Luciferase_2 (F/R):

CACCGACTGTCTGTGGAGATGTGCA\AAACTGCACATCTCCACAGACAGTC

PPP2R2A_1 (F/R):

CACCGTGTCATCTTTCAACAGGAGC\AAACGCTCCTGTTGAAAGATGACAC

PPP2R2A_2 (F/R):

CACCGGAGGAGGGAATGATATTCAG\AAACCTGAATATCATTCCCTCCTCC

PPP2R5C_1 (F/R):

CACCGTACGGGAGCGGAATTTGACC\AAACGGTCAAATTCCGCTCCCGTAC

PPP2R5C_2 (F/R):

CACCGTGTACCGCAACTCAAAGACC\AAACGGTCTTTGAGTTGCGGTACAC

PPP2R5D_1 (F/R):

CACCGCCTTCATCGAATCCCACAGG\AAACCCTGTGGGATTCGATGAAGGC

PPP2R5D_2 (F/R):

CACCGCAGTGAGGATCCTCGAGAGC\AAACGCTCTCGAGGATCCTCACTGC

PPP2R5E_1 (F/R):

CACCGGTTGGTGTACAATGTGTTGA\AAACTCAACACATTGTACACCAACC

PPP2R5E_2 (F/R):

CACCGTGTTTGACGAGCTGACAGCC\AAACGGCTGTCAGCTCGTCAAACAC

Intracranial Xenografts

Approximately 1×10^5 cells (3 μL) were intracranially injected into the brain of athymic nu/nu female mice (6–8 weeks old). Mice implanted with U87-MG cells were treated with 10 mg/kg perphenazine for 5 days followed by 2 days off, starting eight days post implantation. Mice implanted with U87-MG-PTEN cells received ten total treatments of 10 mg/kg perphenazine for 5 days followed by 2 days off, starting eight days post implantation. Mice were monitored for weight loss or severe neuro-symptoms until moribund, at which point they were sacrificed.

Bioluminescence Imaging

Mice implanted with U87-MG-Luc2 cells were intraperitoneally injected with a luciferin (PerkinElmer: 122796) solution (15 mg/ml in PBS, dose of 150 mg/kg). Bioluminescence images were acquired using an IVIS Lumina system and analyzed by Living Image software.

MTS Viability Assays

Cells were seeded in a 96-well plate at a concentration of 1×10^4 cells per well. Drugs/controls were added 24 hours after plating. 48 hours later, 20 μL/well MTS reagent (abcam: ab197010) was added to each well, incubate for 1 hour at 37 °C in standard culture conditions and measured.

Immunofluorescence

About $1.5x10^4$ cells were plated per well in an 8-well chamber slide and incubated overnight. Cells were washed 2x with ice-cold PBS and fixed with 4% formaldehyde for 10 minutes. Cells were washed 2x with ice-cold PBS and permeabilized using 0.5% triton X-100 in PBS. Cells were incubated in blocking buffer (5% horse serum, 2% fish gelatin) for 60 minutes at 37 °C. Primary antibody was added in blocking buffer and incubated overnight at 4 °C on a rocker. Slides were washed 3x with PBST (0.1% Tween 20) and Invitrogen IgG (H+L) Highly Cross-Adsorbed Secondary Antibody was added at 1:1000 in blocking buffer. Cells were incubated for 1 hour at 37 °C and washed 1x with PBST. Slides were mounted with ProLong Diamond Antifade Mountant with DAPI (Invitrogen: P36971) and sealed. Imaging was conducted on Cytation 5 imager.

NAD^+/NADH Quantification

NAD^+/NADH quantification was performed using the NAD^+/NADH Quantification Kit (Sigma: MAK037). Approximately $2x10^5$ cells were seeded per well in a 6-well plate. Cells were incubated with drugs for 24 hours prior to cell lysis. Cells were pelleted, washed with PBS, and lysed in 500 μL extraction buffer. We homogenized the lysates using ten rounds of sonication (1s, 10% amplitude, with a 30s interval, Branson 450 Digital Sonifier). 250 μL of each sample was aliquoted and incubated in a heating block at 60 °C for 30 minutes to decompose NAD^+. For each sample, 50 μL of total (NAD^+ & NADH) and NADH alone (decomposed NAD^+) were plated in triplicate along with the provided NADH standard. 100 μL reaction mix containing the NAD^+ cycling enzyme was added to all samples for 5 minutes to convert NAD^+ to NADH. Subsequently, we added 10 μL of the NADH

developing reagent to each sample and incubated at room temperature for 90 minutes. Absorbance was read using a Cytation 5 imager (Biotek). NAD^+ levels were determined by subtracting NADH from decomposed samples from total NADH.

Seahorse XF Real-Time ATP Rate Assay Kit

We assessed glycolytic and mitochondrial ATP production using the Seahorse XF Real-Time ATP Rate Assay Kit (Agilent: 103592-100). 10,000 cells were plated per well in a 24-well plate, 24 hours prior to starting the assay. 1 hour before starting the experiment, the cell culture media was changed out with Seahorse XF DMEM medium (Agilent: 103680-100) containing 10 mM glucose, 1 mM pyruvate, 2 mM glutamine, and desired vehicle or drugs. The assay was run on an XFe24 analyzer using the standard Seahorse XF Real-Time ATP Rate Assay protocol (20 min: 1.5 μM oligomycin; 40 min: 0.5μM Rotenone/antimycin A).

Triple Quad LC-MS/MS

Metabolites were quantified using the multiple reactions monitoring method (MRM) by selecting the appropriate qualifier and quantifier using a Triple Quad LC-MS/MS (Agilent). The source parameters were as follows: drying gas temperature 325 °C, drying gas flow 11 L/min, nebulizer gas pressure 45 psi, and capillary voltage 4000 V. The MS scan was fixed at 100-1000 mass-to-charge ratio (m/z), and the data scan speed was fixed at 1-spectra per second for data acquisition. Samples was injected on a reverse-phase C18 stable bond column (2.1 x 50 mm x 1.8 μm) (Agilent: 827700-902). Mobile phase consisted of water containing 0.1% acetic acid (A) and ACN containing 0.1% acetic acid (B). The samples were resolved for 12.5 min at a flow rate of 0.4 mL/min and the column

temperature was maintained at 60 °C. The mobile phase solvent gradient consisted of 100% ‘A’ for 0.5 min with ramp of curve 0-45% B from 0.5 min to 4.5 min then 45-90% ‘B’ from 4.5 min to 7.5 min and hold at 90% ‘B’ from 7.5 min to 9.5 min. Further, the gradient was brought back to 100% ‘A’ from 9.5 to 9.6 min then held at 100% ‘A’ from 9.6 min to 12.5 min. To optimize compound identification, we used the Mass Hunter Optimizer program (Agilent) to find the optimum collision energy, fragmentor voltage, precursor, and production ions using spectroscopic grade synthetic compounds. Consequently, compound retention time was determined. Standard curves were generated using spectroscopic grade synthetic compounds of target metabolites. 5 μL of each sample was injected for analysis and the metabolite abundance was determined using the corresponding standard curve. The retention time, fragmentation pattern, quantifier ion and qualifier ion for each compound are provided in Supplemental Figure 5.

Supplemental Figure Legends:

Supplemental Figure 1: The cytotoxic effect of perphenazine is independent of DRD2 antagonism.

A) Microscopic images of empty vector (EV) and PTEN overexpression U87-MG cells treated with vehicle or 10 μM perphenazine for 48 hours.

B) Images showing $PI(3,4,5)P_3$ and $PI(4,5)P_2$ levels in T98G cells treated with vehicle or 10 μM perphenazine for 48 hours.

C) Assessment of DRD2 protein levels using western blot of GBM cell lines.

D) Viability of T98G cells 48 hours following the indicated treatments.

E) Western blot analysis of T98G cells transfected with 100 nM siDRD2 for 48 hours.

F) Viability of T98G cells transfected with siDRD2 48 hours following the indicated treatments.

G) DEMETER2 dependency scores of all neurotransmitter receptor genes across GBM cell lines. siDRD1 is indicated in red.

H) Chronos dependency scores of all neurotransmitter receptor genes across GBM cell lines. sgDRD1 and sgDRD2 are indicated in red.

Supplemental Figure 2: Inhibition of PP2A does not rescue the anti-GBM activity of perphenazine.

A) Relative viability of U87-MG cells treated with perphenazine and/or LB-100 for 48 hours.

B) Top: Relative viability of U87-MG cells treated with perphenazine and/or 10 nM siPPP2CA for 48 hours. Bottom: Assessment of indicated proteins using western blot of U87-MG cells treated with 10 μM perphenazine and/or 10 nM siPPP2CA for 48 hours.

C) Top: Relative viability of U87-MG cells overexpressing EV or SV40 ST protein and/or perphenazine for 48 hours. Bottom: Assessment of indicates protein levels in U87-MG cells transfected with EV or SV40 ST expression plasmids.

D) Assessment of indicated protein levels using western blot of proteins labeled via biotinylation by the myc-BioID2-PP2A-Aα fusion protein and isolated using streptavidin beads from T98G and U87-MG cells.

E) Assessment of indicated protein levels using western blot of proteins from U87-MG cells treated with perphenazine for 24 hours.

F) Assessment of indicated protein levels using western blot of proteins from U87-MG transduced with CRISPR-Cas9 transduction particles with sgRNAs targeting the indicated genes.

G) Relative viability of the indicated U87-MG isogenic cell lines in response to PPZ.

Supplemental Figure 3: Perphenazine blocks autophagic flux.

A) U87-MG cells stained with LipidTox 4 hours following treatment with vehicle or 10 μM perphenazine.

B) Representative images of lysotracker stained cells 1 hour post treatment with 10 μM perphenazine. Scale bar: 20 μm.

Assessment of indicated protein levels using western blot of U87-MG cells treated with vehicle or 10 μM perphenazine and grown in C) glucose deprived, D) glutamine deprived, or E) serum depleted conditions for 24 hours. Relative viability of U87-MG cells over 72 hours following treatment with vehicle or 10 μM perphenazine and grown in F) glucose deprived, G) glutamine deprived, or H) serum depleted conditions.

I) Representative images of proximity ligation assays of the indicated proteins in U87-MG cells 24 hours after treatment with vehicle or 10 μM perphenazine. Scale bar: 5 μm.

Supplemental Figure 4: Perphenazine blocks endocytic flux.

A) Assessment of indicated protein levels using western blot of T98G cells treated with 10 μM perphenazine for the indicated amounts of time.

B) Representative images of EGFR and TfR localization using immunofluorescent staining. Scale bar: 5 μm.

C-D) Representative images immunofluorescence assays targeting the indicated proteins in U87-MG cells 24 hours after treatment with vehicle or 10 μM perphenazine. Scale bar: 5 μm.

E-F) Representative images of proximity ligation assays of the indicated proteins in U87-MG cells 24 hours after treatment with vehicle or 10 μM perphenazine. Scale bar: 5 μm.

Supplemental Figure 5: Inhibitors of apoptosis, necroptosis, ferroptosis, and autophagy do not rescue the cytotoxicity of perphenazine.

A) Viability of U87-MG cells 24 hours after addition of 15 ng/mL TNFα +/- 1 hour pre-treatment with the indicated doses of necrostatin-1.

B) Viability of U87-MG cells 48 hours after addition of the indicated doses of perphenazine +/- 1 hour pre-treatment with the indicated doses of necrostatin-1.

C) Viability of U87-MG cells 24 hours after addition of 2 μM staurosporine +/- 1 hour pre-treatment with the indicated doses of V-ZAD-FMK.

D) Viability of U87-MG cells 48 hours after addition of perphenazine +/- 1 hour pre-treatment with the indicated doses of V-ZAD-FMK.

E) Viability of U87-MG cells 24 hours after addition of 10 μM erastin +/- 1 hour pre-treatment with the indicated doses of ferrostatin-1.

F) Viability of U87-MG cells 48 hours after addition of perphenazine +/- 1 hour pre-treatment with the indicated doses of ferrostatin-1.

G) Viability of U87-MG cells transfected with siATG5, siATG7, and ATG4B (C74A) 48 hours after addition of vehicle or 10 μM perphenazine.

H) Annexin V / PI analysis of U87-MG cells treated with vehicle or 10 μM perphenazine for 24 hours.

Supplemental Figure 6: LC-MS/MS retention time, quantifier and qualifier ions for MRM quantification of metabolites.

A) Western blot analysis of immuno-purified vesicles used in downstream LC-MS/MS analysis.

B) Viability of the indicated cell lines following treatment with CQ for 48 hours.

C-D) Ion mode, quantifier and qualifier ions, retention time (RT), and spectrum of compounds quantified using LC-MS/MS.
