# Supplementary material for: Antipsychotics possess anti-glioblastoma activity by disrupting lysosomal function and inhibiting oncogenic signaling by stabilizing PTEN
Source: Cell Death Dis. 2024 Jun 13;15(6):414. doi: 10.1038/s41419-024-06779-3 (PMC11176297; doi:10.1038/s41419-024-06779-3)
Supplement: Supplementary file 2 — Supplemental Figure 1 to Supplemental Figure 6 [file 41419_2024_6779_MOESM2_ESM.pptx]

## Slide 1
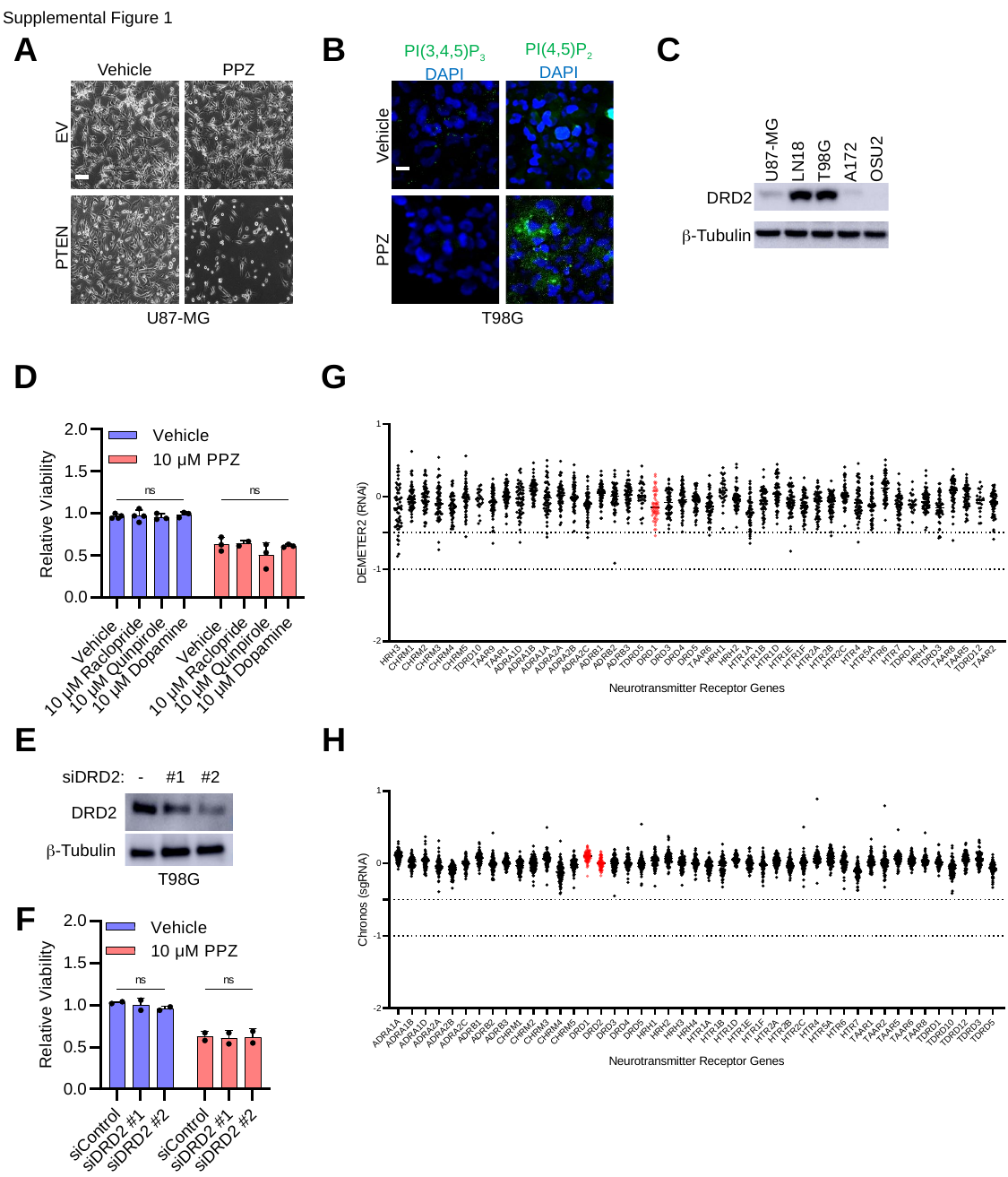

Supplemental Figure 1
A
B
C
PI(4,5)P2
DAPI
PI(3,4,5)P3
DAPI
Vehicle
PPZ
U87-MG
LN18
T98G
A172
OSU2
DRD2
b-Tubulin
EV
Vehicle
PTEN
PPZ
U87-MG
T98G
D
G
E
H
siDRD2:
-
#1
#2
DRD2
b-Tubulin
T98G
F

## Slide 2
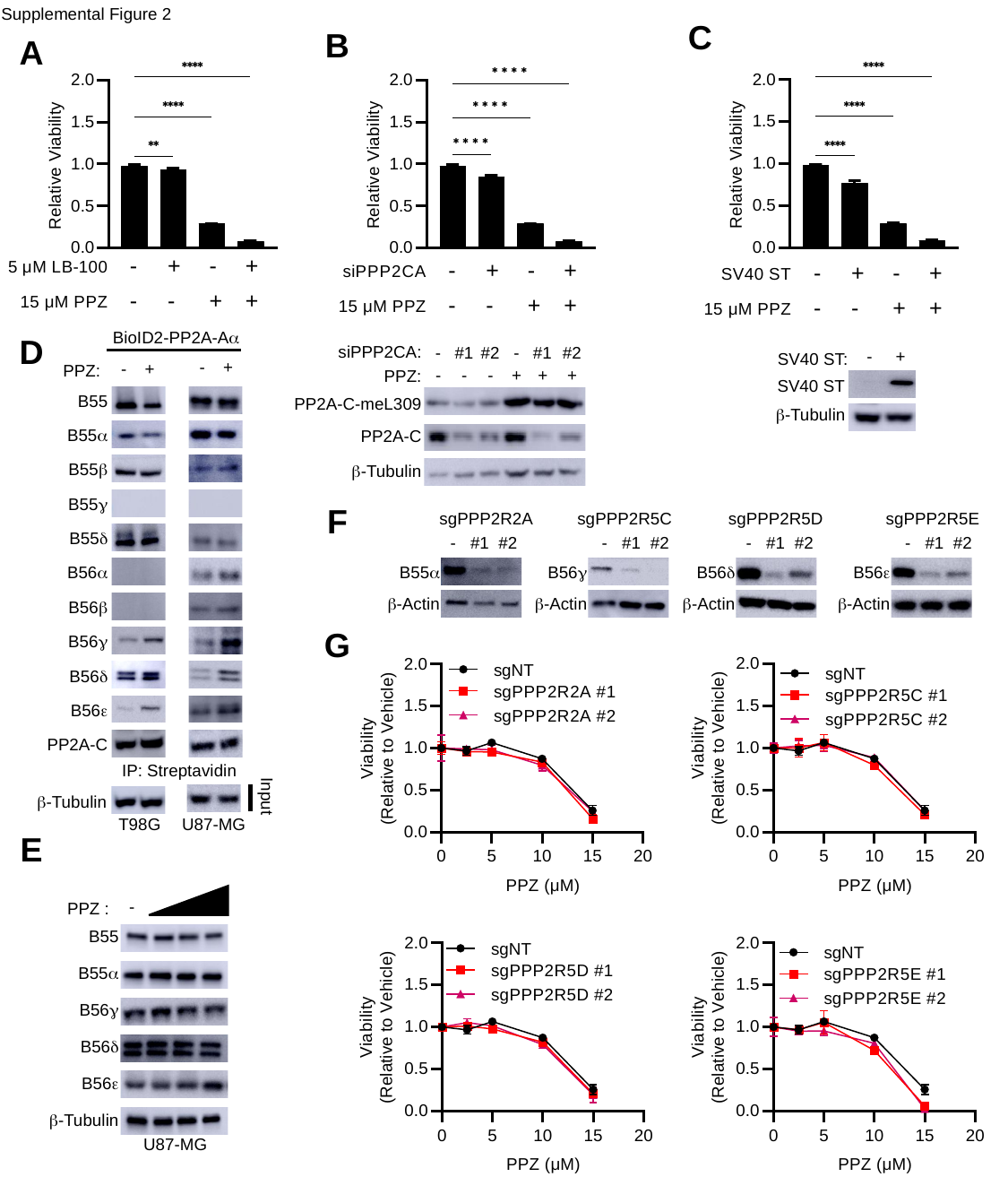

Supplemental Figure 2
C
B
A
BioID2-PP2A-Aa
-
+
-
+
PPZ:
B55
B55a
B55b
B55g
B55d
B56a
B56b
B56g
B56d
B56e
PP2A-C
IP: Streptavidin
Input
b-Tubulin
T98G
U87-MG
D
siPPP2CA:
-
#1
#2
-
#1
#2
+
-
SV40 ST:
-
-
-
+
+
+
PPZ:
SV40 ST
PP2A-C-meL309
b-Tubulin
PP2A-C
b-Tubulin
F
sgPPP2R2A
sgPPP2R5C
sgPPP2R5D
sgPPP2R5E
-
#1
#2
-
#1
#2
-
#1
#2
-
#1
#2
B55a
B56g
B56d
B56e
b-Actin
b-Actin
b-Actin
b-Actin
G
E
-
PPZ :
B55
B55a
B56g
B56d
B56e
b-Tubulin
U87-MG

## Slide 3
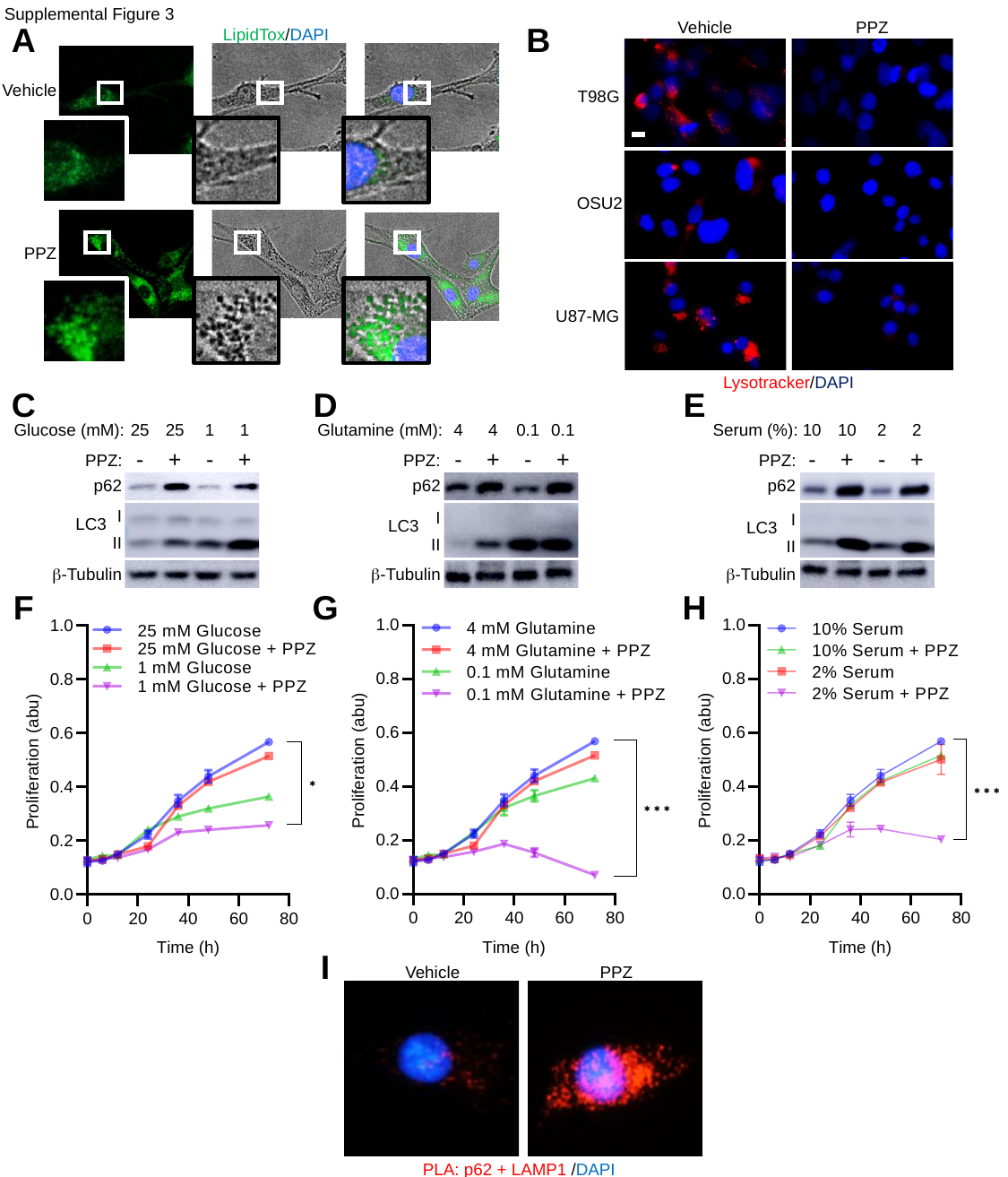

Supplemental Figure 3
Vehicle
PPZ
T98G
OSU2
U87-MG
Lysotracker/DAPI
A
B
LipidTox/DAPI
Vehicle
PPZ
C
D
E
Glucose (mM):
25
25
1
1
Glutamine (mM):
4
4
0.1
0.1
Serum (%):
10
10
2
2
-
+
-
+
-
+
-
+
-
+
-
+
PPZ:
PPZ:
PPZ:
p62
p62
p62
I
I
I
LC3
LC3
LC3
II
II
II
b-Tubulin
b-Tubulin
b-Tubulin
F
G
H
I
PPZ
Vehicle
PLA: p62 + LAMP1 /DAPI

## Slide 4
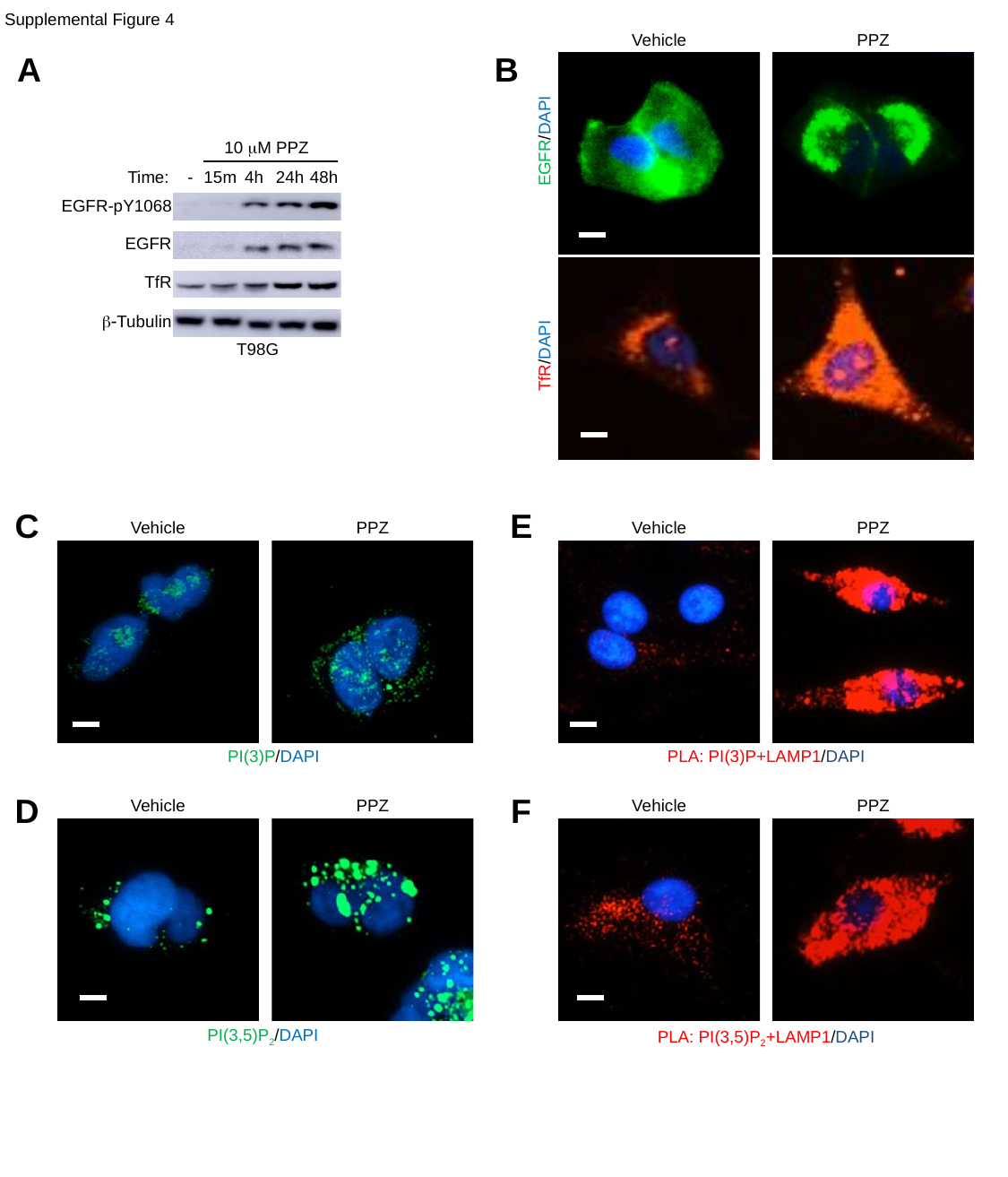

Supplemental Figure 4
Vehicle
PPZ
A
B
EGFR/DAPI
10 mM PPZ
Time:
-
15m
4h
24h
48h
EGFR-pY1068
EGFR
TfR
b-Tubulin
T98G
TfR/DAPI
C
E
Vehicle
PPZ
Vehicle
PPZ
PI(3)P/DAPI
PLA: PI(3)P+LAMP1/DAPI
D
F
Vehicle
PPZ
Vehicle
PPZ
PI(3,5)P2/DAPI
PLA: PI(3,5)P2+LAMP1/DAPI

## Slide 5
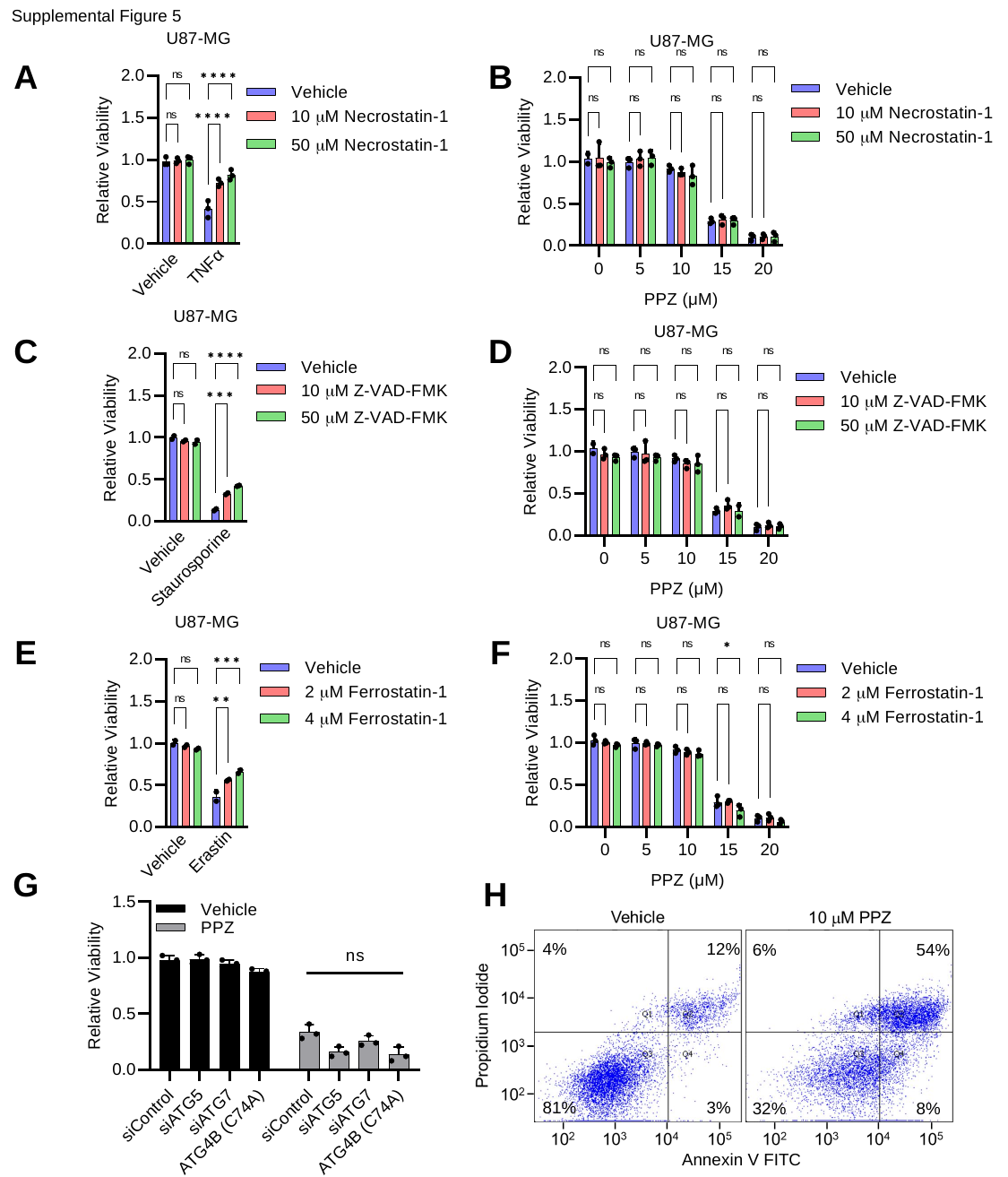

Supplemental Figure 5
A
B
C
D
E
F
G
H
Annexin V FITC
4%
12%
6%
54%
81%
3%
32%
8%

## Slide 6
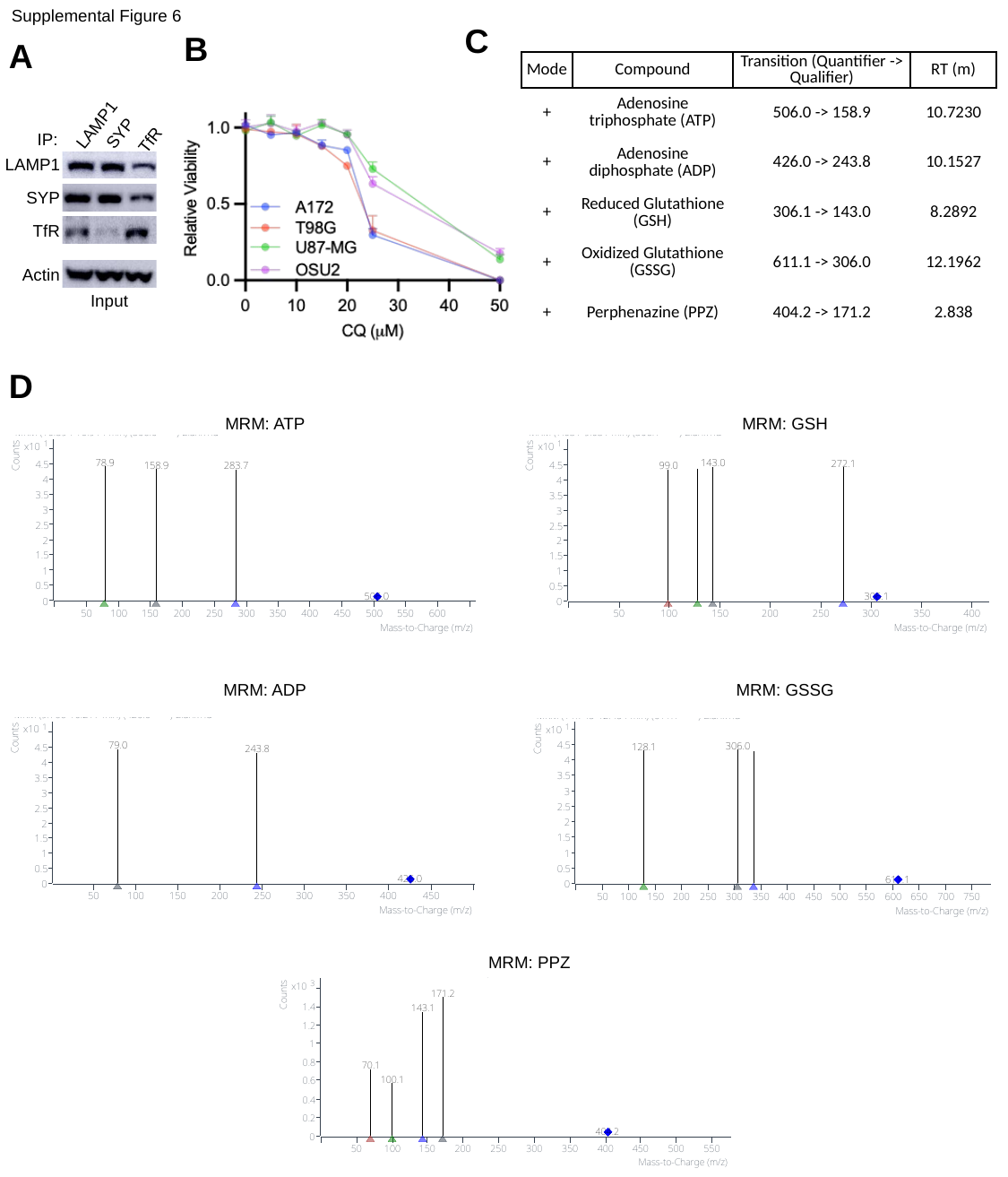

Supplemental Figure 6
C
B
A
| Mode | Compound | Transition (Quantifier -> Qualifier) | RT (m) |
| --- | --- | --- | --- |
| + | Adenosine triphosphate (ATP) | 506.0 -> 158.9 | 10.7230 |
| + | Adenosine diphosphate (ADP) | 426.0 -> 243.8 | 10.1527 |
| + | Reduced Glutathione (GSH) | 306.1 -> 143.0 | 8.2892 |
| + | Oxidized Glutathione (GSSG) | 611.1 -> 306.0 | 12.1962 |
| + | Perphenazine (PPZ) | 404.2 -> 171.2 | 2.838 |
LAMP1
SYP
TfR
IP:
LAMP1
SYP
TfR
Actin
Input
D
MRM: ATP
MRM: GSH
MRM: ADP
MRM: GSSG
MRM: PPZ
