# Supplementary material for: Antipsychotics possess anti-glioblastoma activity by disrupting lysosomal function and inhibiting oncogenic signaling by stabilizing PTEN
Source: Cell Death Dis. 2024 Jun 13;15(6):414. doi: 10.1038/s41419-024-06779-3 (PMC11176297; doi:10.1038/s41419-024-06779-3)
Supplement: Supplementary file 4 — Raw western blot images [file 41419_2024_6779_MOESM4_ESM.pptx]

## Slide 1
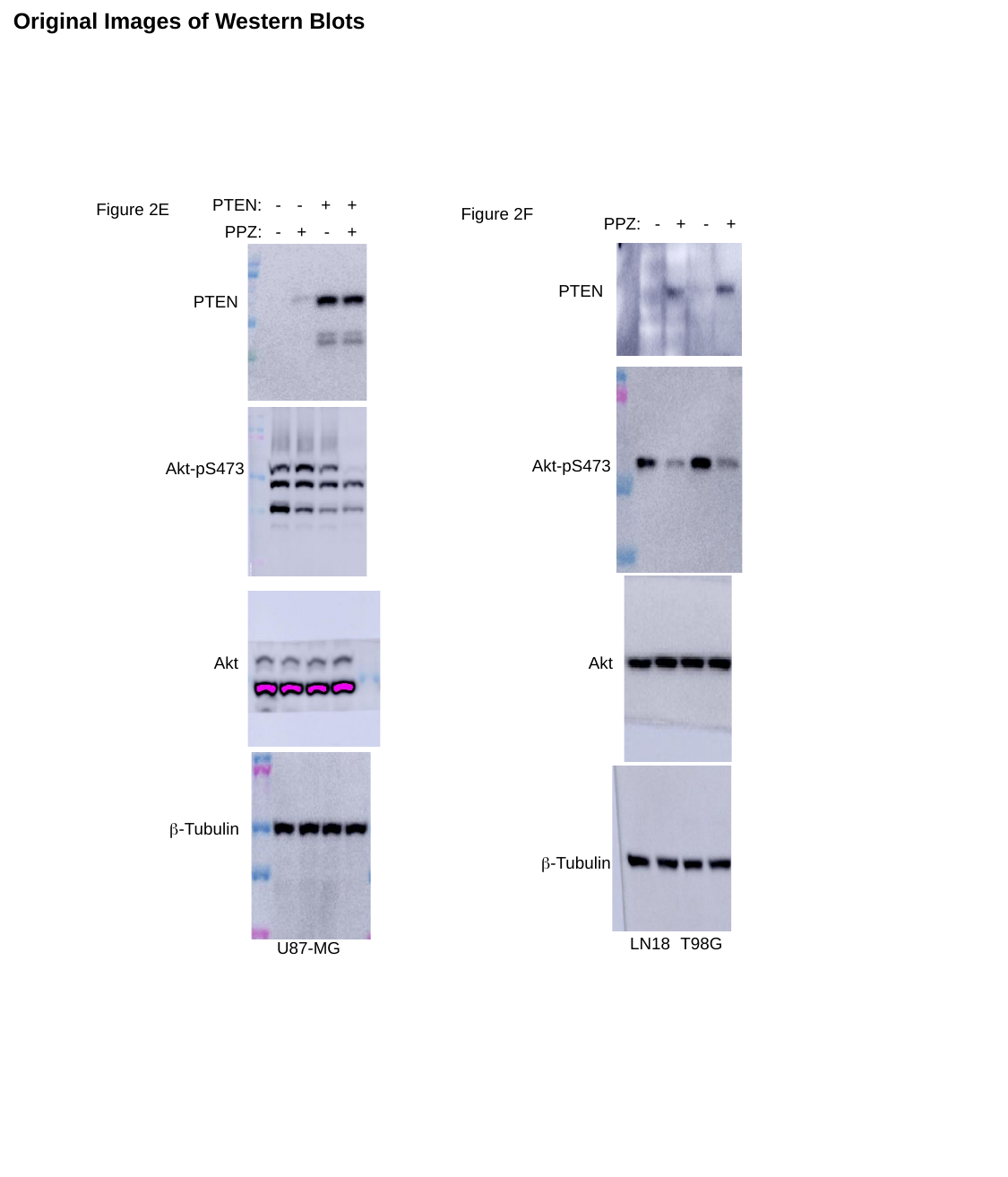

Original Images of Western Blots
PTEN:
-
-
+
+
Figure 2E
Figure 2F
PPZ:
-
+
-
+
PPZ:
-
+
-
+
PTEN
PTEN
Akt-pS473
Akt-pS473
Akt
Akt
b-Tubulin
b-Tubulin
LN18
T98G
U87-MG

## Slide 2
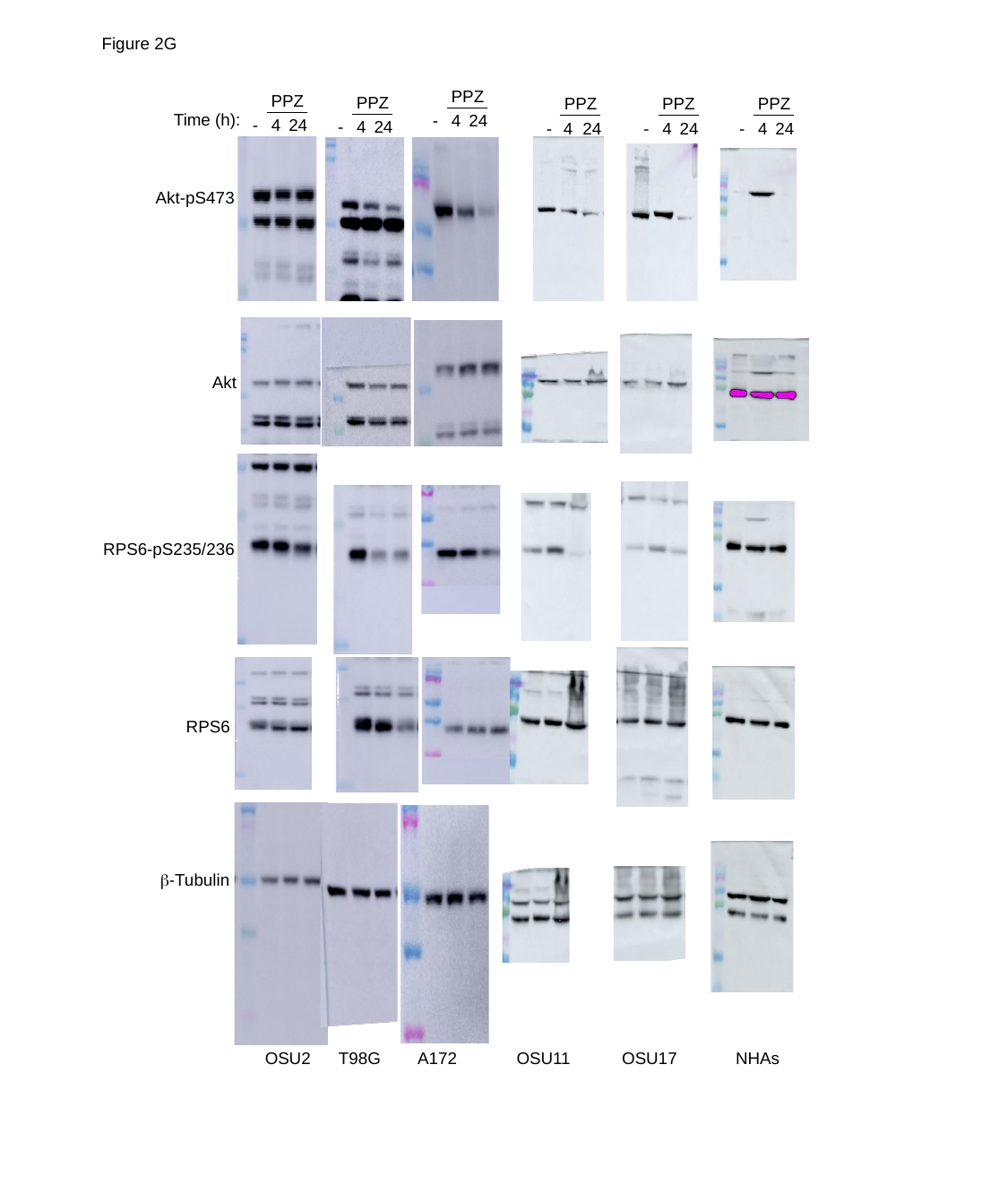

Figure 2G
PPZ
-
4
24
PPZ
-
4
24
PPZ
-
4
24
PPZ
PPZ
PPZ
Time (h):
-
4
24
-
4
24
-
4
24
Akt-pS473
Akt
RPS6-pS235/236
RPS6
b-Tubulin
OSU2
T98G
A172
OSU11
OSU17
NHAs

## Slide 3
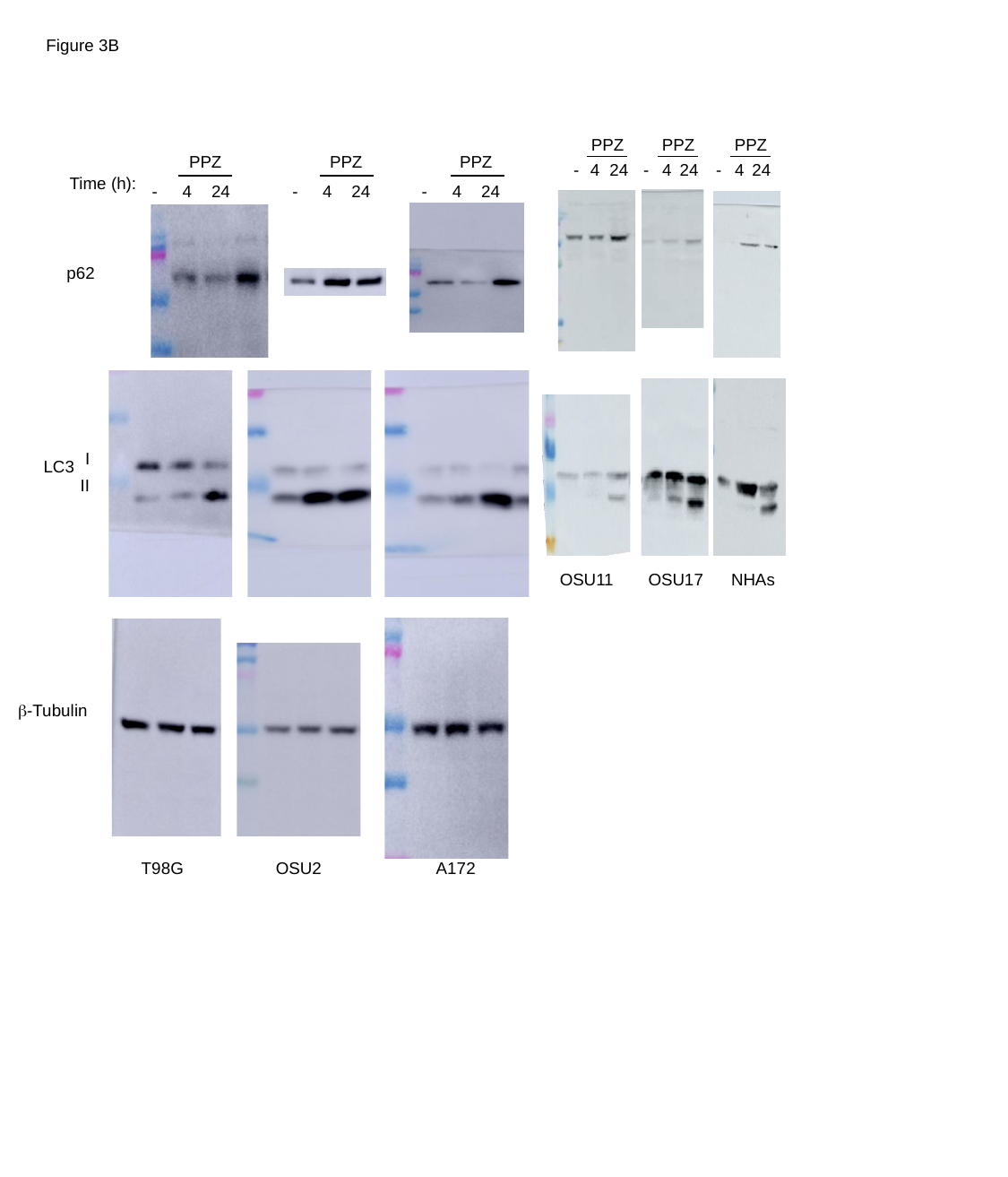

Figure 3B
PPZ
PPZ
PPZ
PPZ
PPZ
PPZ
-
4
24
-
4
24
-
4
24
Time (h):
-
4
24
-
4
24
-
4
24
p62
I
LC3
II
OSU11
OSU17
NHAs
b-Tubulin
T98G
OSU2
A172

## Slide 4
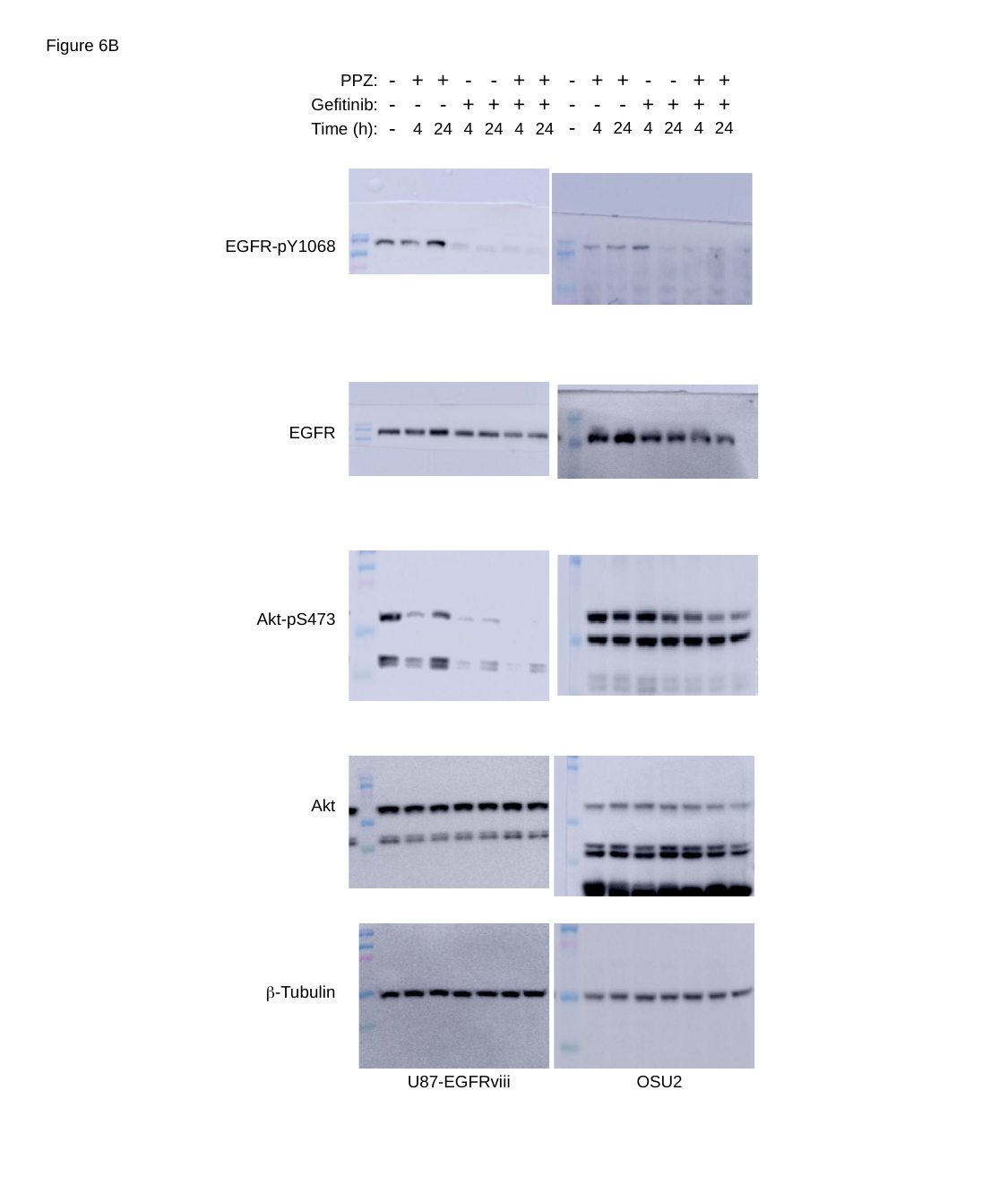

Figure 6B
-
+
+
-
-
+
+
-
+
+
-
-
+
+
PPZ:
-
-
+
+
+
+
-
-
-
+
+
+
+
-
Gefitinib:
-
-
4
24
4
24
4
24
4
24
4
24
4
24
Time (h):
EGFR-pY1068
EGFR
Akt-pS473
Akt
b-Tubulin
U87-EGFRviii
OSU2

## Slide 5
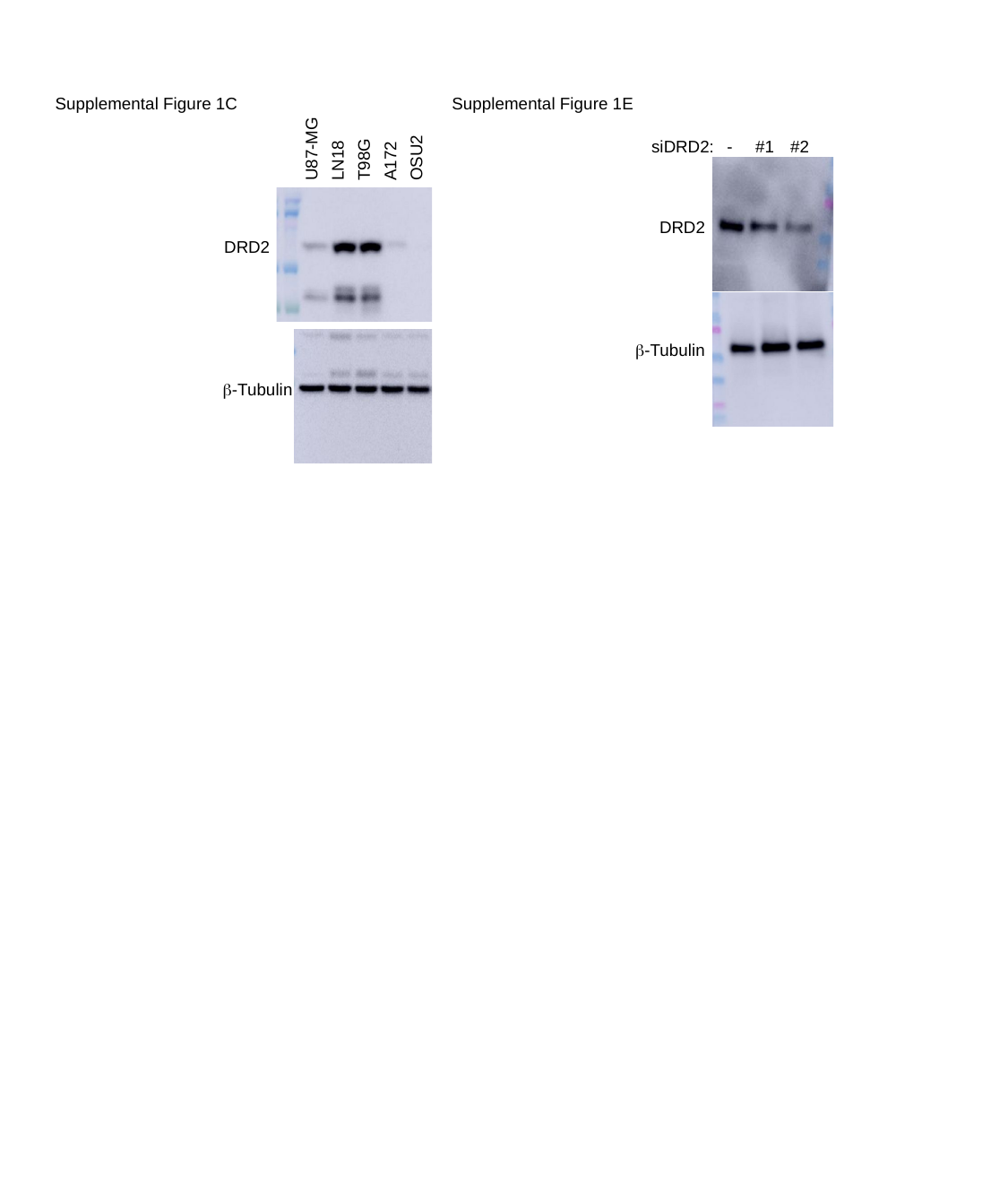

Supplemental Figure 1C
Supplemental Figure 1E
U87-MG
LN18
T98G
A172
OSU2
siDRD2:
-
#1
#2
DRD2
DRD2
b-Tubulin
b-Tubulin

## Slide 6
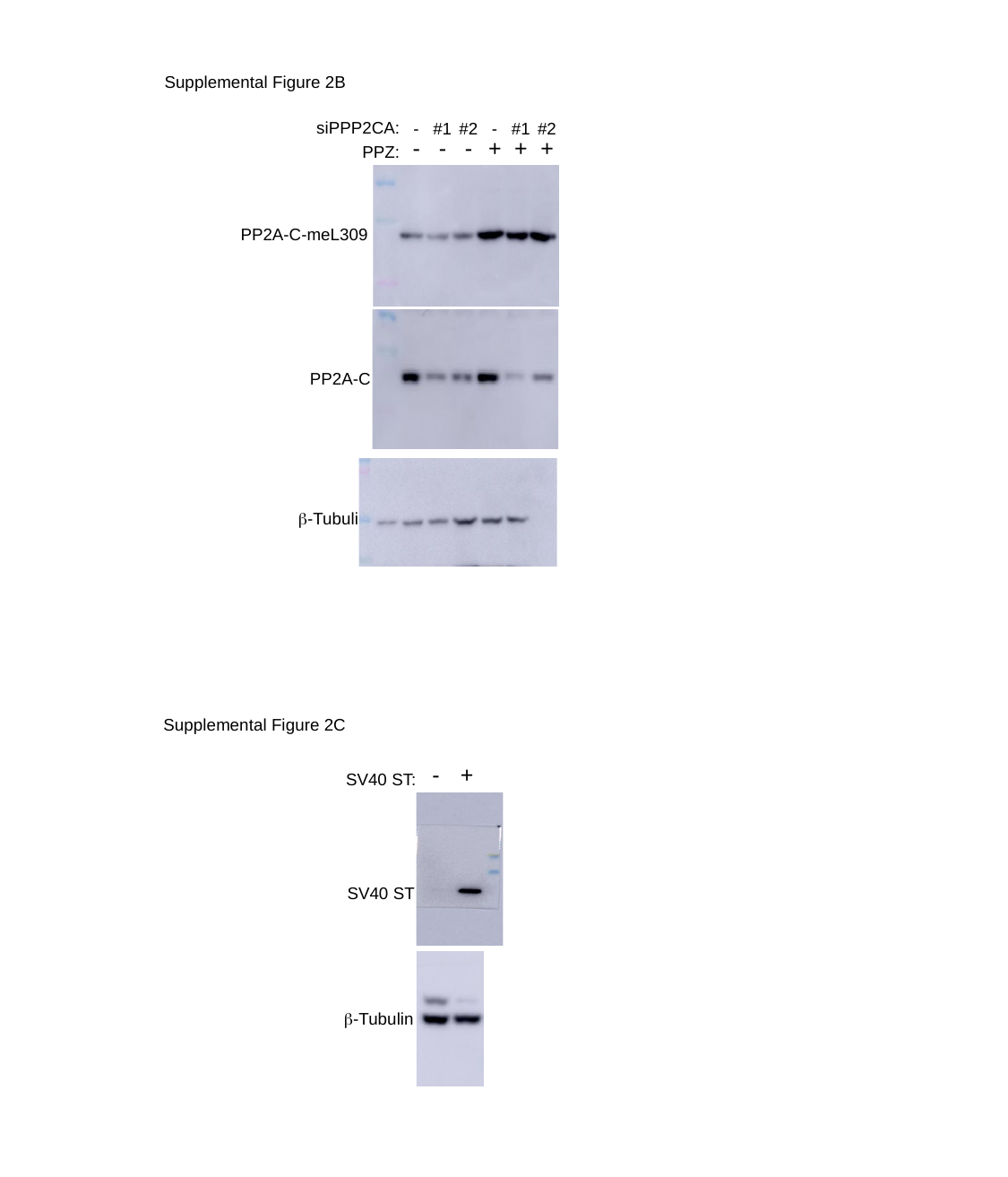

Supplemental Figure 2B
siPPP2CA:
-
#1
#2
-
#1
#2
-
-
-
+
+
+
PPZ:
PP2A-C-meL309
PP2A-C
b-Tubulin
Supplemental Figure 2C
-
+
SV40 ST:
SV40 ST
b-Tubulin

## Slide 7
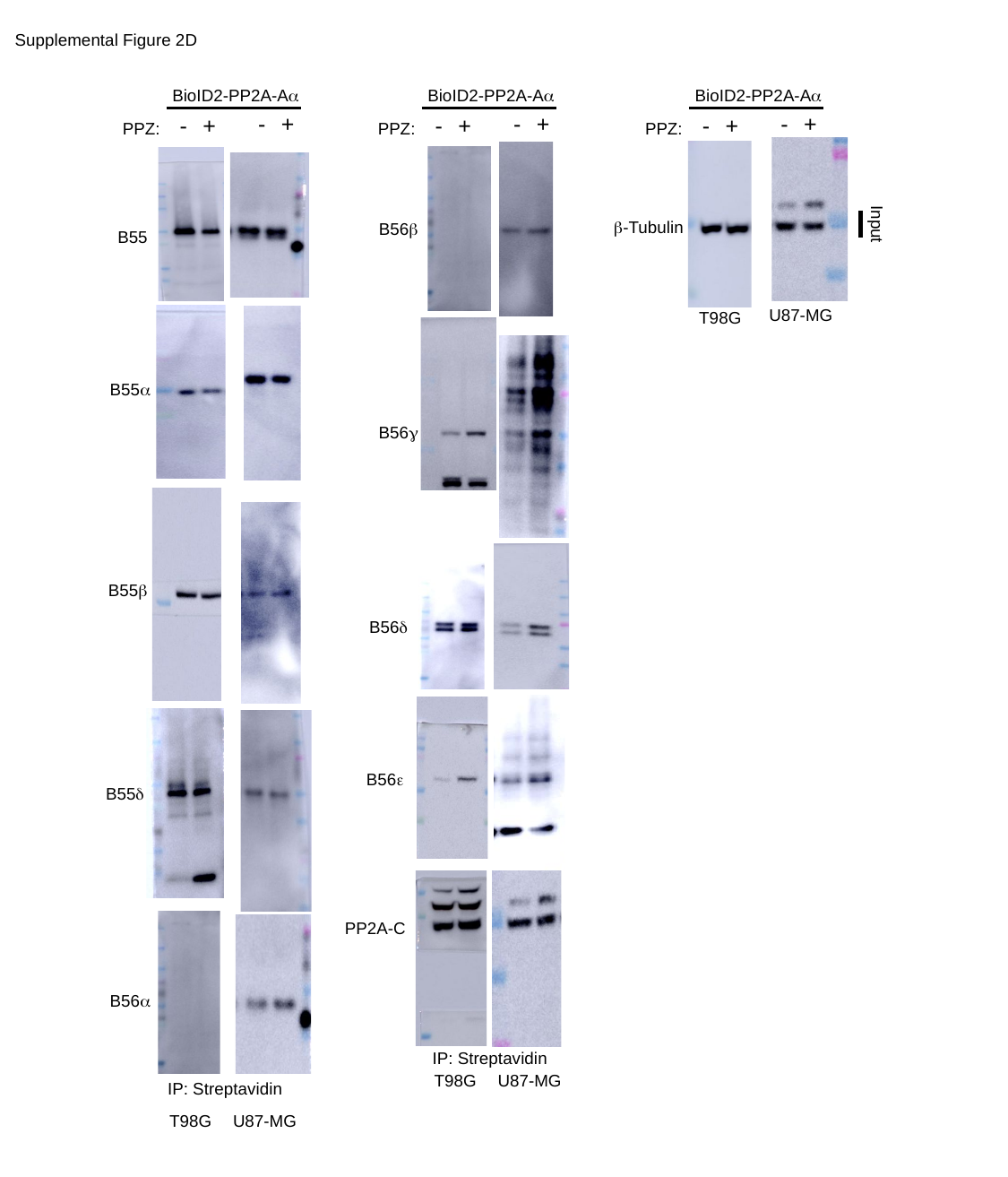

Supplemental Figure 2D
BioID2-PP2A-Aa
-
+
-
+
PPZ:
BioID2-PP2A-Aa
-
+
-
+
PPZ:
BioID2-PP2A-Aa
-
+
-
+
PPZ:
Input
b-Tubulin
B56b
B55
U87-MG
T98G
B55a
B56g
B55b
B56d
B56e
B55d
PP2A-C
B56a
IP: Streptavidin
T98G
U87-MG
IP: Streptavidin
T98G
U87-MG

## Slide 8
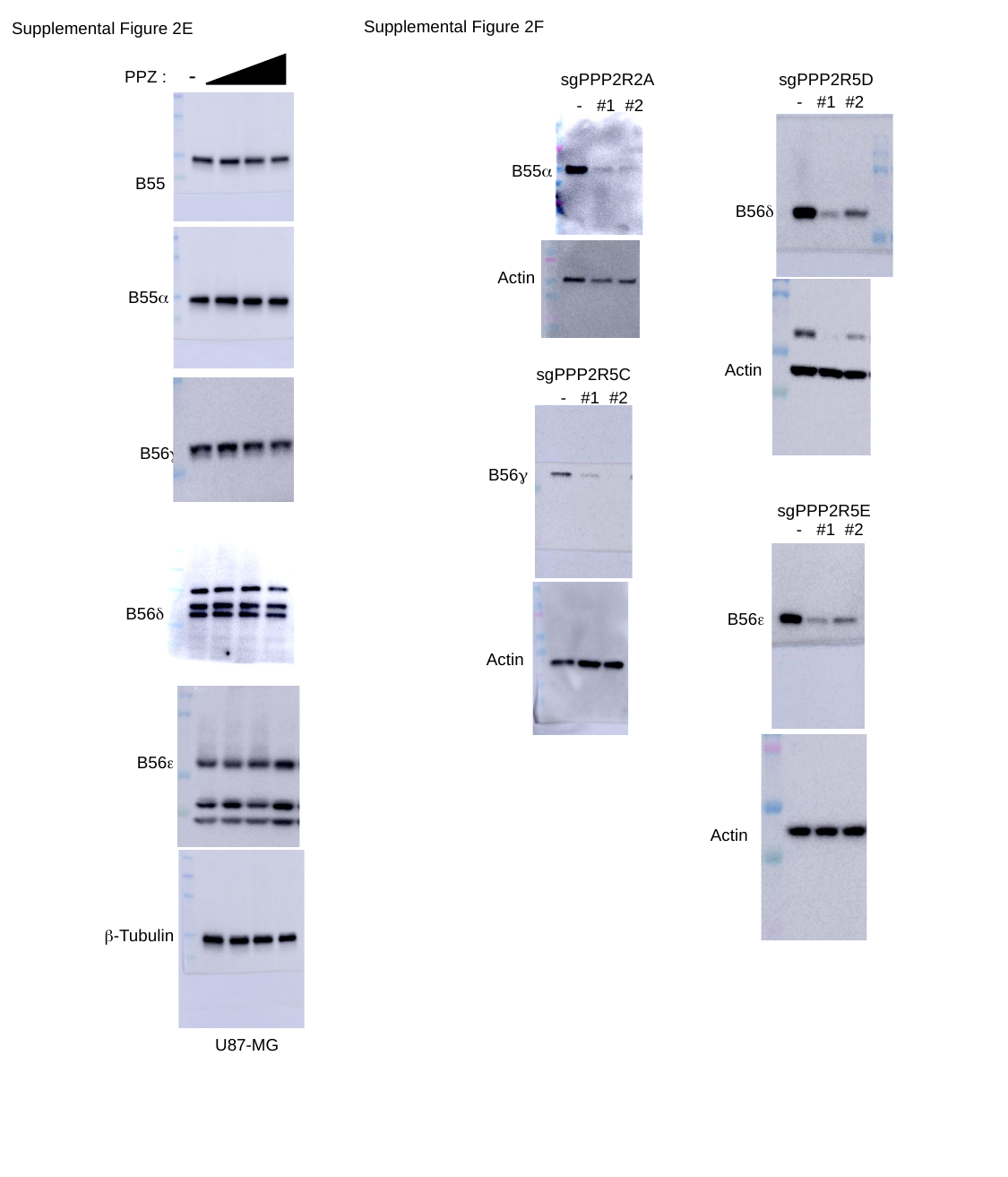

Supplemental Figure 2F
Supplemental Figure 2E
-
PPZ :
sgPPP2R2A
sgPPP2R5D
-
#1
#2
-
#1
#2
B55a
B55
B56d
Actin
B55a
Actin
sgPPP2R5C
-
#1
#2
B56g
B56g
sgPPP2R5E
-
#1
#2
B56d
B56e
Actin
B56e
Actin
b-Tubulin
U87-MG

## Slide 9
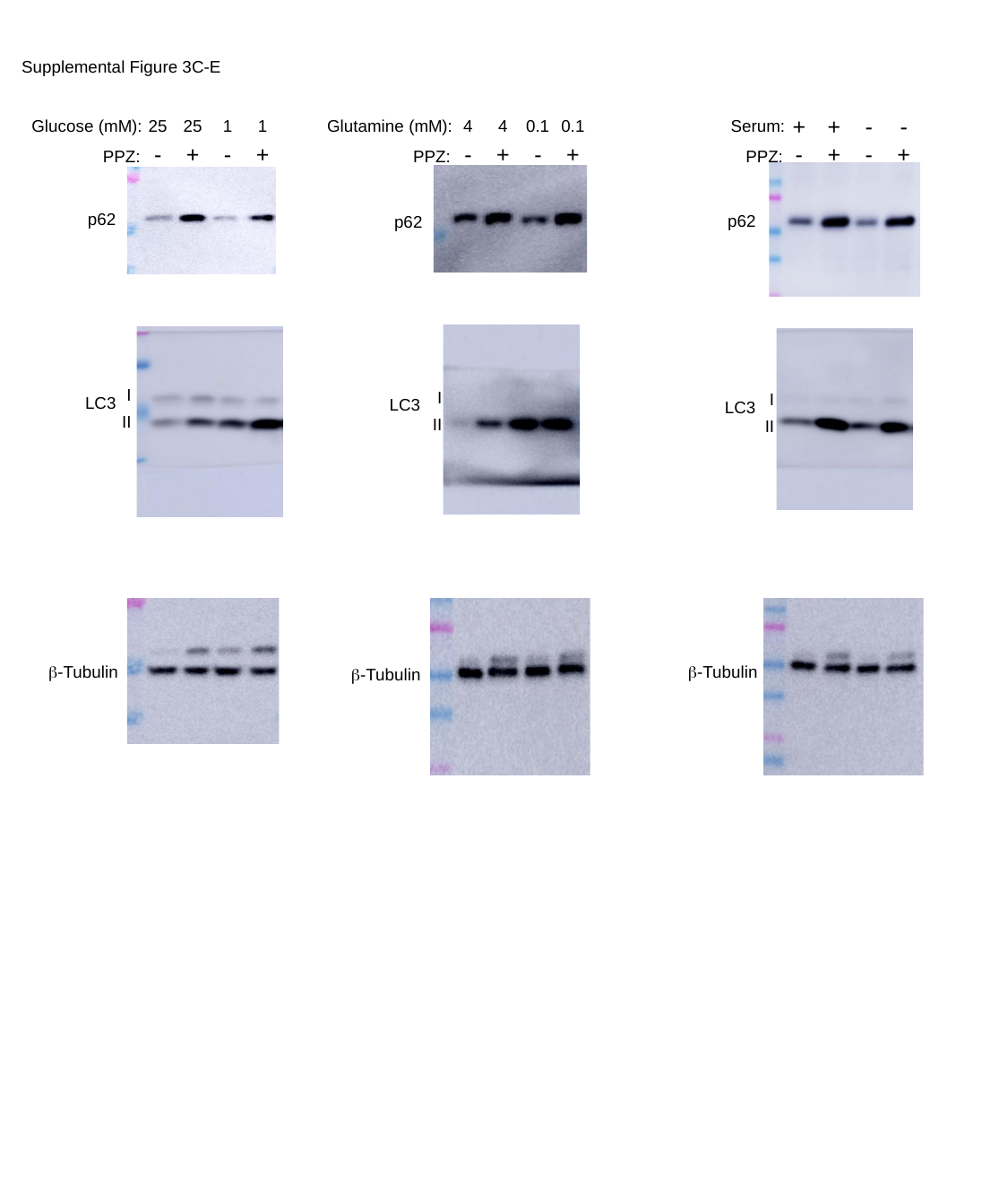

Supplemental Figure 3C-E
+
+
-
-
Glucose (mM):
25
25
1
1
Glutamine (mM):
4
4
0.1
0.1
Serum:
-
+
-
+
-
+
-
+
-
+
-
+
PPZ:
PPZ:
PPZ:
p62
p62
p62
I
I
I
LC3
LC3
LC3
II
II
II
b-Tubulin
b-Tubulin
b-Tubulin

## Slide 10
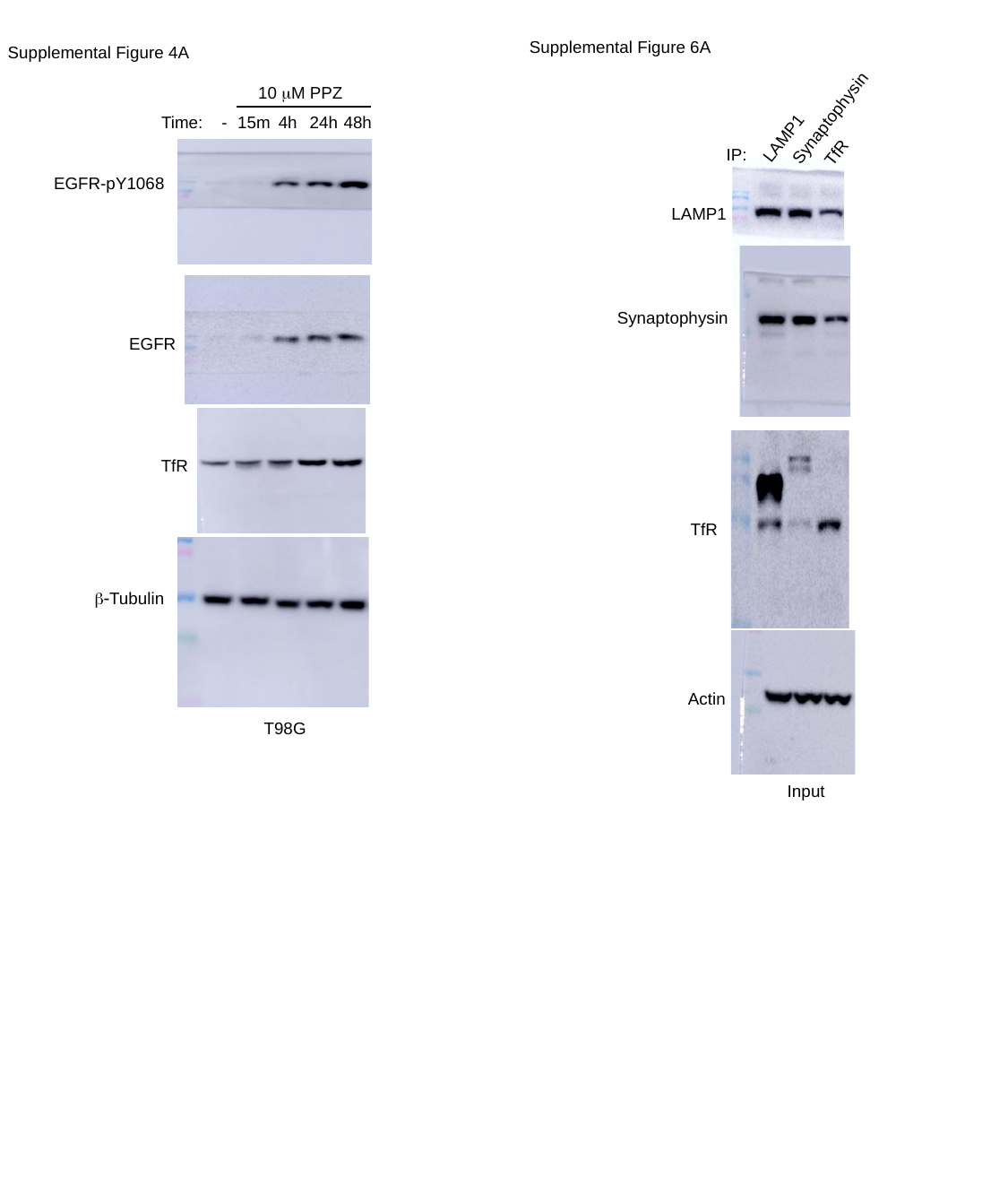

Supplemental Figure 6A
Supplemental Figure 4A
10 mM PPZ
Synaptophysin
LAMP1
TfR
Time:
-
15m
4h
24h
48h
IP:
EGFR-pY1068
LAMP1
Synaptophysin
EGFR
TfR
TfR
b-Tubulin
Actin
T98G
Input
